# Structural basis for human Ca_v_3.2 inhibition by selective antagonists

**DOI:** 10.1038/s41422-024-00959-8

**Published:** 2024-04-11

**Authors:** Jian Huang, Xiao Fan, Xueqin Jin, Chen Lyu, Qinmeng Guo, Tao Liu, Jiaofeng Chen, Amaël Davakan, Philippe Lory, Nieng Yan

**Affiliations:** 1 Department of Molecular Biology, Princeton University, Princeton, NJ USA; 2 Beijing Frontier Research Center for Biological Structures, State Key Laboratory of Membrane Biology, Tsinghua-Peking Joint Center for Life Sciences, School of Life Sciences, Tsinghua University, Beijing, China; 3 IGF, Université de Montpellier, CNRS, INSERM, LabEx ‘Ion Channel Science and Therapeutics’, Montpellier, France; 4 Institute of Bio-Architecture and Bio-Interactions, Shenzhen Medical Academy of Research and Translation, Shenzhen, Guangdong China; 5 Present Address: Laboratory of Neurophysiology and Behavior, The Rockefeller University, New York, NY USA

**Keywords:** Cryoelectron microscopy, Molecular modelling

## Abstract

The Ca_v_3.2 subtype of T-type calcium channels has been targeted for developing analgesics and anti-epileptics for its role in pain and epilepsy. Here we present the cryo-EM structures of Ca_v_3.2 alone and in complex with four T-type calcium channel selective antagonists with overall resolutions ranging from 2.8 Å to 3.2 Å. The four compounds display two binding poses. ACT-709478 and TTA-A2 both place their cyclopropylphenyl-containing ends in the central cavity to directly obstruct ion flow, meanwhile extending their polar tails into the IV-I fenestration. TTA-P2 and ML218 project their 3,5-dichlorobenzamide groups into the II-III fenestration and place their hydrophobic tails in the cavity to impede ion permeation. The fenestration-penetrating mode immediately affords an explanation for the state-dependent activities of these antagonists. Structure-guided mutational analysis identifies several key residues that determine the T-type preference of these drugs. The structures also suggest the role of an endogenous lipid in stabilizing drug binding in the central cavity.

## Introduction

Voltage-gated calcium (Ca_v_) channels respond to membrane depolarization, allowing Ca^2+^ influx and translating electrical signals into intracellular Ca^2+^-mediated events.^1–4^ They play instrumental roles in Ca^2+^-dependent physiological processes such as neurotransmitter/hormone release, gene expression, and muscle contraction.^3,5–9^ Precise control of calcium channel activity involves various cellular factors such as receptors, calmodulin, phospholipids, and G proteins.^10–18^ Additionally, these channels can be modulated by a range of ions, toxins, and numerous clinical and investigational drugs.^19–24^

Among the 10 mammalian Ca_v_ subtypes (Ca_v_1.1-Ca_v_1.4, Ca_v_2.1-Ca_v_2.3, and Ca_v_3.1-Ca_v_3.3), the Ca_v_3 subfamily, also known as the T-type calcium channels, is characterized by tiny and transient currents, in contrast to the large and long-lasting L-type Ca_v_1 channels.^25–28^ Unlike Ca_v_1 and Ca_v_2 members, which require multiple auxiliary subunits for channel activities, the T-type channels function independently.^29–31^ In addition, while Ca_v_1 and Ca_v_2 channels are activated at high voltage, thus also known as high voltage-activated (HVA) channels, Ca_v_3 channels are low voltage-activated (LVA).^28,32,33^ Their functional distinctions are supported by a large degree of sequence variations between the LVA and HVA channels, offering an opportunity to develop subtype-specific modulators for potential drug discovery.

Ca_v_3.2, encoded by *CACNA1H* and widely expressed in the central nervous system, regulates neuronal excitability and participates in nociception.^34,35^ Alterations in its activity are associated with many neurological and neuropsychiatric disorders, including idiopathic generalized epilepsy and pain.^36–39^ Studies indicate increased expression and/or activity of Ca_v_3.2 in spinal dorsal horn and in dorsal root ganglion neurons in various inflammatory and neuropathic pain models.^36^ Silencing or pharmacological inhibition of Ca_v_3.2 channels induces analgesia in rodents.^40^ Consequently, Ca_v_3.2 is emerging as a promising drug target for the development of next-generation analgesics. Compounds targeting Ca_v_3.2, such as ethosuximide, mibefradil, valproate, zonisamide, pimozide, and certain dihydropyridines (DHPs), demonstrate efficacy in rodent models of acute, inflammatory, and chronic pain.^40–42^

There are ongoing efforts to scrutinize various small molecules with novel chemical skeletons as potential analgesic candidates, such as Z944, TTA-A2, TTA-P2, ML218, and ACT-709478.^43–46^ Compared to the approved drugs that usually have limited specificity for T-type channels, these investigational T-type-specific blockers demonstrate stronger affinities for T-type Ca_v_ channels. Although these compounds have yet to discriminate between T-type subtypes, the distinctive pharmacokinetic properties of Ca_v_3.2, compared to Ca_v_3.1 and Ca_v_3.3, offer an opportunity to develop bona fide Ca_v_3.2-selective molecules.

Gaining high-resolution insights into the structures of channels complexed with FDA-approved drugs or lead compounds is crucial for guiding de novo drug design or optimization. The structure of Ca_v_3.1 bound to a lead compound Z944 reveals the basic architecture of the T-type channels and the molecular basis for the state-dependent inhibition of Ca_v_3 subfamily by Z944.^44^ A recent report on the structures of Ca_v_3.3 in complex with mibefradil, pimozide, and otilonium bromide broadens the spectrum of drug recognitions targeting T-type calcium channels.^47^ In this study, we sought to determine the structures of human Ca_v_3.2 alone and in complex with representative antagonists, with a particular focus on those exhibiting enhanced selectivity for T-type calcium channels.

## Results

### Structural determination of human Ca_v_3.2

The bottleneck in the structural analysis of human Ca_v_3.2 arises from the low yield of recombinant protein expression. To enhance protein production, various constructs were explored. The I-II loop plays an inhibitory role in Ca_v_3.2 expression and function.^48,49^ We thereby introduced several internal truncations of different fragments to this region. Eventually, a variant with the deletion of residues 493-772 resulted in an elevated expression level and decent solution behavior (Supplementary information, Fig. S1). This variant was named Ca_v_3.2EM, as it was used for cryogenic electron microscopy (cryo-EM) imaging.

**Fig. 1:**
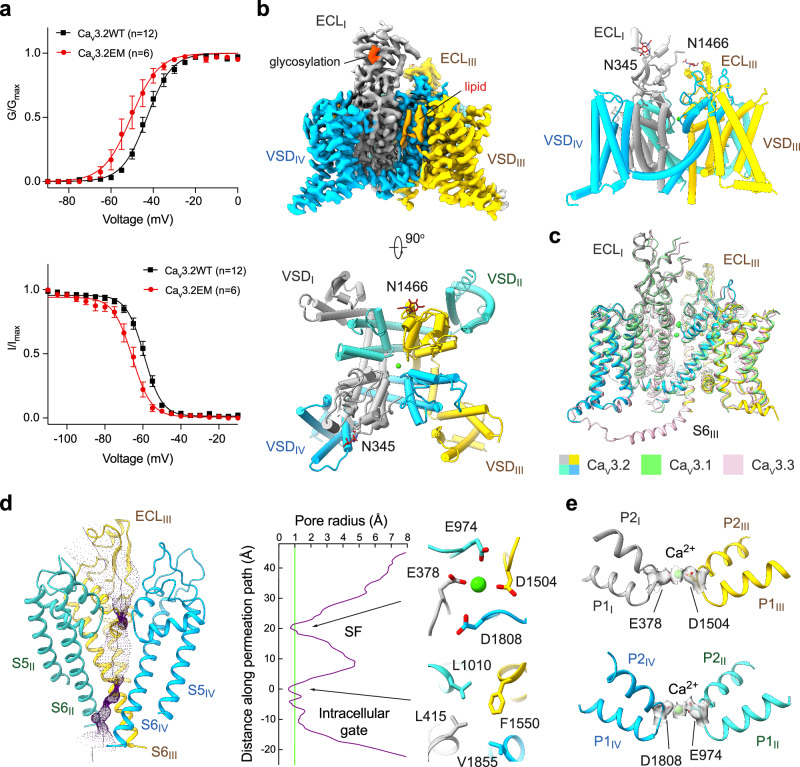
Cryo-EM structural analysis of human Ca_v_3.2. **a** Electrophysiological characterizations of full-length human Ca_v_3.2WT (black) and truncated Ca_v_3.2EM (red) in HEK293T cells. Voltage-dependent activation and steady-state inactivation curves are presented in the upper and lower panels, respectively. *n* values indicate the number of independent cells; data are presented as mean ± SEM. Please refer to Materials and Methods and Supplementary information for details. **b** Cryo-EM reconstruction of human Ca_v_3.2EM. The EM map is color-coded for the four repeats (upper left), and the sugar moieties and lipids are colored bright and pale orange, respectively. The same color scheme is applied throughout the manuscript. The overall structure of human Ca_v_3.2 is shown in a side view (upper right) and top view (bottom). **c** Structural comparison of the three T-type Ca_v_ channels: Ca_v_3.1 (PDB: 6KZO, light green), Ca_v_3.2 (domain colored), and Ca_v_3.3 (PDB: 7WLI, pink). **d** The ion-conducting path of Ca_v_3.2 is calculated in HOLE^70^ and illustrated with purple dots (left). The calculated pore radii along the permeation path are depicted as a purple line (middle). Two constriction sites, the SF enclosed by the EEDD motif and the intracellular gate, are shown on the right, in extracellular views. **e** Ca^2+^ coordination in the SF. Densities for the SF, prepared in ChimeraX, are contoured at the level of 4 σ, and the potential Ca^2+^ ion is shown as a green sphere.

The biophysical properties of Ca_v_3.2EM were verified through whole-cell patch-clamp recordings. Consistent with its improved protein expression, Ca_v_3.2EM exhibited an increased conductance compared with the wild-type (WT) channel. Additionally, both the activation and steady-state inactivation curves of Ca_v_3.2EM demonstrated a slight leftward shift in comparison to WT (Fig. 1a; Supplementary information, Fig. S2 and Table S1).

Following the functional validation of Ca_v_3.2EM, we performed standard cryo-EM single particle analysis and obtained a three-dimensional (3D) EM reconstruction at an overall resolution of 3.0 Å. This structure is henceforth referred to as Ca_v_3.2Apo. Two glycan chains were observed, attaching to Asn345 and Asn1466 in the extracellular loops in repeats I and III (ECL_I_ and ECL_III_) via N-link (Fig. 1b; Supplementary information, Figs. S3, S4 and Table S2). Previous studies have also suggested the importance of these N-glycosylation sites in regulating the functional expression of Ca_v_3.2.^50–52^

The overall structure of Ca_v_3.2Apo displays an inactivated state nearly identical to that observed for Ca_v_3.1 and Ca_v_3.3,^44,47^ with root-mean-square deviations (RMSDs) of 0.58 Å over 847 Cα atoms and 1.16 Å over 948 Cα atoms, respectively (Fig. 1c). All four voltage-sensing domains (VSDs) adopt the depolarized or “up” conformation, and the intracellular gate is tightly twisted. The closed intracellular gate comprises three layers of hydrophobic residues. The first assembly site beneath the central cavity includes conserved residues Leu415, Leu1010, Phe1550, and Val1855, a common feature observed in the structures of all three members of Ca_v_3 channels (Fig. 1d). A spherical density, likely corresponding to a Ca^2+^ ion, is surrounded by four residues, Glu378, Glu974, Asp1504, and Asp1808 (the EEDD motif) in the selectivity filter (SF) (Fig. 1e).

### Structural determination of Ca_v_3.2 with different antagonists

Next, we set out to determine the structures of Ca_v_3.2 in complex with representative T-type channel-selective antagonists. To validate the action of these compounds on both WT Ca_v_3.2 channel and Ca_v_3.2EM variant, we conducted whole-cell patch-clamp recordings in HEK293T cells (Supplementary information, Fig. S5 and Tables S3, S4). The characterizations indicated that Ca_v_3.2EM exhibited comparable potency across all tested compounds compared to the WT channel. Each antagonist was individually incubated with purified Ca_v_3.2 protein at a final concentration at least 10-fold higher than its IC_50_ value before cryo-sample preparation. Following similar protocols for cryo-EM data acquisition and analysis, we successfully resolved the structures of Ca_v_3.2 in complex with four compounds, ACT-709478, TTA-A2, TTA-P2, and ML218, with overall resolutions ranging from 2.8 Å to 3.2 Å. For simplicity, we will refer to these structures as Ca_v_3.2-ACT/TA/TP/ML, with “ACT” representing ACT-709478, “TA” for TTA-A2, “TP” for TTA-P2, and “ML” for ML218 (Supplementary information, Fig. S6 and Table S2).

All four complex structures resemble the apo form, with the compounds each accommodated within the pore domain (PD) (Supplementary information, Fig. S6q). The four compounds can be further categorized into two groups: ACT-709478 and TTA-A2 insert through the IV-I fenestration, while TTA-P2 and ML218 dock on the II-III fenestration. Apart from the fenestration binding site, the other end of these elongated compounds is nestled within the central cavity. The fenestration-accommodating binding poses immediately suggest the molecular basis for their state-dependent pore-blocking mechanism. In the following text, we will illustrate their binding details, which will facilitate future drug design and optimization.

### Coordination of ACT-709478 and TTA-A2

Both ACT-709478 and TTA-A2 were well resolved. The distinct structural features within the densities, like the trifluoromethyl group in ACT-709478 and the methyl group in TTA-A2, enabled reliable model building of these small-molecule compounds (Supplementary information, Fig. S6).

**Fig. 2:**
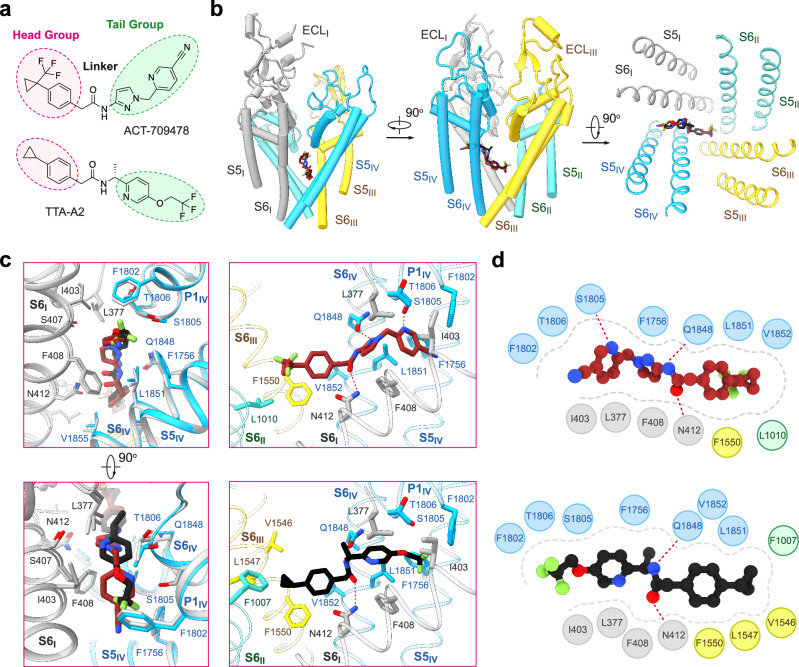
Molecular basis for Ca_v_3.2 inhibition by ACT-709478 or TTA-A2. **a** Chemical structures of ACT-709478 and TTA-A2. The two compounds share a common core structure, featuring a cyclopropylphenyl head (highlighted in red), an aromatic tail (highlighted in green), and an amide linker. **b** Structural basis for pore block by ACT-709478 or TTA-A2. Both molecules traverse the central cavity, with one end inserting into the IV-I fenestration. The two structures, named Ca_v_3.2-ACT and Ca_v_3.2-TA, are superimposed relative to the PD. ACT-709478 and TTA-A2 are shown as brown and black sticks, respectively, and only the PD of Ca_v_3.2-ACT is shown. **c** Detailed coordination of ACT-709478 and TTA-A2. The aromatic tail groups of ACT-709478 and TTA-A2, which vary in details, can both be accommodated in the IV-I fenestration. Potential H-bonds are highlighted with red dashed lines. **d** Schematic representation of residues constituting the binding site for ACT-709478 (upper) and TTA-A2 (lower). Residues within a 4 Å cutoff distance to the ligand are shown, with the binding pocket and potential H-bonds indicated by gray dashed contour and red dashed lines, respectively.

The head groups, characterized by the cyclopropylphenyl group in TTA-A2 or the trifluoromethyl cyclopropylphenyl moiety in ACT-709478, reside in the center of the cavity, which we named site C in Na_v_ channels^53,54^ (Fig. 2a, b). These groups are coordinated similarly by several hydrophobic residues from the S6 tetrahelical bundles, including Phe408 and Asn412 on S6_I_, Phe1007 and Leu1010 on S6_II_, Val1546, Leu1547, and Phe1550 on S6_III_, and Gln1848, Leu1851, and Val1852 on S6_IV_ (Fig. 2c, d).

Both tail groups, despite their distinct chemical structures, wedge into the IV-I fenestration, indicating the fenestration’s versatile adaptability to diverse molecules. Per our recently proposed nomenclature system for the druggable sites on Na_v_ channels,^53^ this site will be described as site F4. The tail groups in the IV-I fenestration are surrounded by hydrophobic residues, Leu377, Ile403, Ser407, and Phe408 from repeat I, as well as Phe1756, Phe1802, Ser1805, and Thr1806 from repeat IV. In the case of ACT-709478, the nitrogen within the cyanopyridine ring is further stabilized through a hydrogen bond (H-bond) with the hydroxyl group from Ser1805. Furthermore, both internal amide linkages are H-bonded with Asn412 and Gln1848, contributing to the stability of these unique binding poses (Fig. 2c, d).

### An α-to-π transition of S6_II_ in the presence of TTA-A2

**Fig. 3:**
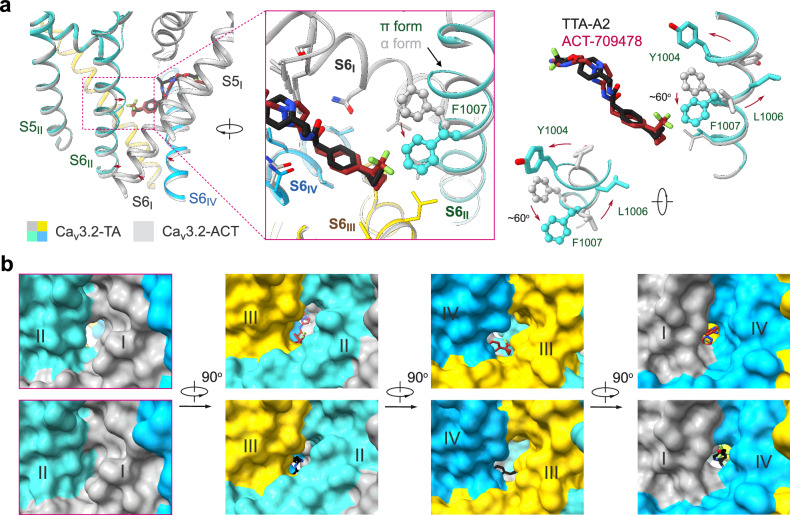
Local structural shifts in the presence of TTA-A2. **a** An α-to-π transition observed in the S6_II_ segment upon TTA-A2 binding. Left: Red arrows indicate the structural differences between Ca_v_3.2-ACT (gray) and Ca_v_3.2-TA (domain colored). An enlarged view that highlights the change of S6_II_ is shown in the inset. Right: Rotation of the bottom half of the S6_II_ helix in the presence of TTA-A2, but not ACT-709478. **b** Closure of the I-II fenestration in Ca_v_3.2-TA. Corresponding surface views of the four sides of the PD are presented for Ca_v_3.2-ACT (upper) and Ca_v_3.2-TA (lower).

ACT-709478 and TTA-A2 share a similar pharmacophore featuring a cyclopropylphenyl head, an aromatic tail, and an amide linkage. However, there are local structural variations in the presence of these two compounds. While the conformation of Ca_v_3.2-ACT is nearly identical to that of the apo channel, structural rearrangements occur in the presence of TTA-A2, as exemplified by an α-to-π transition in the middle of the S6_II_ segment (Fig. 3a).

A close structural comparison of Ca_v_3.2-ACT/TA reveals that the conformational deviation is caused by the minor difference in the head group. The only divergence of the head group of the two molecules pertains to the presence of an additional trifluoromethyl group in ACT-709478. This protrusion would clash with Phe1007 in a π-helical configuration but align well with the α-helix. Yet, Phe1007 provides a favorable environment for accommodating the smaller cyclopropylphenyl group in TTA-A2, explaining the π form of S6_II_ in Ca_v_3.2-TA (Fig. 3a). As a result of the minor rotation of Phe1007, the I-II fenestration, present in the apo channel and Ca_v_3.2-ACT, diminishes in Ca_v_3.2-TA, and the gating residue on S6_II_ shifts from Leu1010 to Val1011 in Ca_v_3.2-TA, with the intracellular gate remaining closed (Figs. 1d, 3b; Supplementary information, Fig. S7).

### TTA-P2 and ML218 bind through the II-III fenestration

**Fig. 4:**
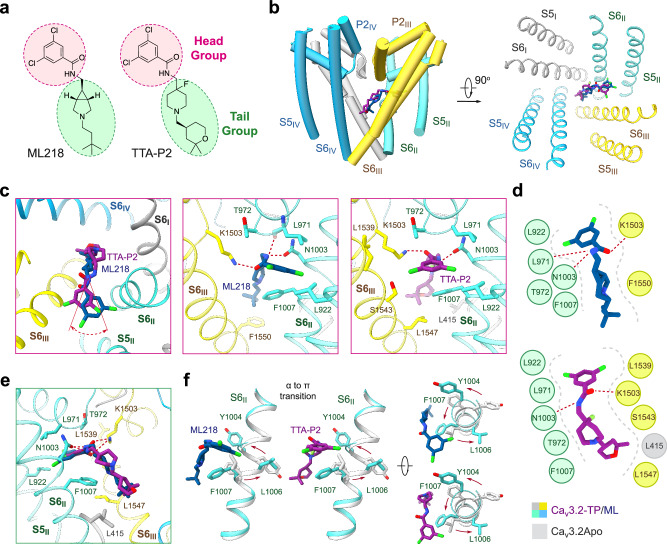
Specific inhibition of Ca_v_3.2 by ML218 or TTA-P2. **a** Chemical structures of ML218 and TTA-P2. **b** The two molecules exhibit similar binding poses. Structures of Ca_v_3.2-ML (with ML218) and Ca_v_3.2-TP (with TTA-P2) are superimposed relative to the PD. Only the PD structure of Ca_v_3.2-ML is shown as semi-transparent cartoon. **c** The binding poses of ML218 and TTA-P2 deviate with an ~30° rotation of the 3,5-dichlorobenzamide head within the II-III fenestration. Detailed coordination of ML218 and TTA-P2 in the II-III fenestration are presented in the middle and right panels, respectively. **d** Schematic representation of residues constituting the binding site for ML218 or TTA-P2 within a 4-Å cutoff distance. The binding pocket and potential H-bonds are indicated by a gray dashed contour and red dashed lines, respectively. **e** Coordination of the aliphatic tail of the two compounds in the central cavity of Ca_v_3.2. **f** Both structures adopt a π configuration in the middle of the S6_II_ segment.

TTA-P2 and ML218 also share a similar chemical structure, characterized by a 3,5-dichlorobenzamide head and an aliphatic tail (Fig. 4a). They also display a similar binding paradigm, with the head adhering to the II-III fenestration (Site F2), and the tail projecting into the cavity (site C) (Fig. 4b). Despite an ~30° deviation of the binding poses for the two head groups, the accommodation site within the fenestration is similar. The environment is primarily hydrophobic, enclosed by residues from S5_II_, P1_II_, S6_II_, and S6_III_ segments, including Leu922 on S5_II_, Leu971 on P1_II_, Asn1003 and Phe1007 on S6_II_, Lys1503 in the P-loop, and Leu1539, Ser1543, Leu1547, and Phe1550 on S6_III_ (Fig. 4c, d).

The aliphatic tail of both TTA-P2 and ML218 reclines across the central cavity, directly obstructing ion permeation. The cavity site is formed by Leu415 on S1_I_, Phe1007 on S6_II_, and Ser1543, Leu1547, and Phe1550 on S6_III_ (Fig. 4d, e) Similar to the conformation observed in the presence of TTA-A2, S6_II_ adopts a π-helix conformation upon binding to TTA-P2 or ML218. On one end, the 3,5-dichlorobenzamide head group would encounter spatial repulsion with Leu1006 in an α-helix conformation; on the other end, the aliphatic tail could be further stabilized by Phe1007 through a π-H interaction, thus favoring the π-helix form (Fig. 4f).

### Stabilization of antagonist binding by an endogenous lipid

**Fig. 5:**
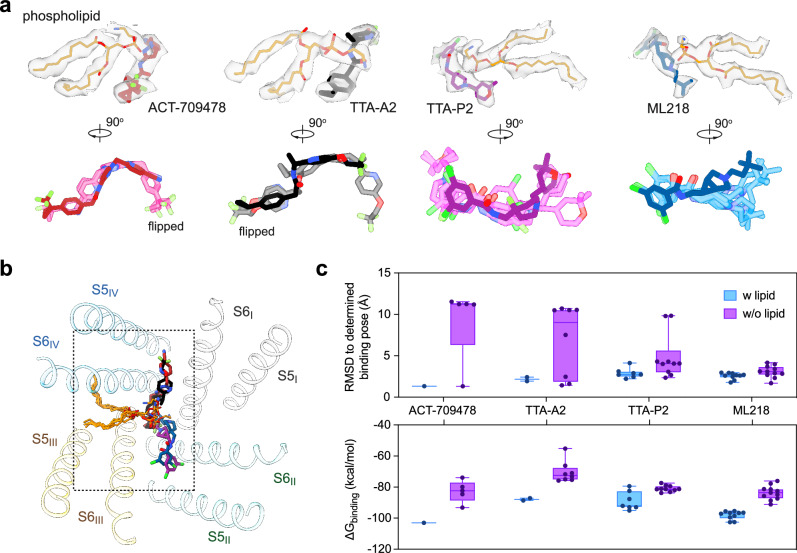
Stabilization of antagonist binding by endogenous lipids. **a** A conserved endogenous lipid stabilizes antagonist association within the central cavity. The EM densities for the lipid and antagonist are contoured at the similar level of 4.5–5 σ. The antagonists show more diverse docking poses in the absence of the endogenous lipid. Predicted binding poses in the absence of the lipid are represented as semi-transparent sticks, while the structure-determined binding pose is shown as solid sticks. **b** Identical binding pose of the lipid in all blocker-bound Ca_v_3.2 structures. Only the pore-forming helices of Ca_v_3.2-TA are shown in an extracellular view of the superimposed structures of the antagonist-bound pore cavity. **c** Less favored binding of antagonists to Ca_v_3.2 in the absence of the lipid. Predicted binding poses in the absence of the endogenous lipid exhibit larger variability, as indicated by the increased RMSD values. The average binding free energy (ΔG_binding_) was calculated using Prime-MM/GBSA.

Examination of the 3D EM maps for all the structures, including that of the apo channel, identifies a well-resolved density in the cavity, which can be best fitted with a phosphatidylethanolamine (PE) molecule (Fig. 5a, b; Supplementary information, Fig. S8). The two hydrophobic tails penetrate the III-IV fenestration, and the polar head inserts into the central cavity, directly contributing to drug coordination. Similar paradigm has been observed in drug-bound structures of Ca_v_1.1, Ca_v_3.1, and Ca_v_3.3.^22,44,47^

To investigate the role of the lipid in drug binding, we performed molecular docking simulations with or without the lipid. The docking poses in the presence of the lipid align well with the experimental structures, with RMSD values of the predicted binding poses < 3.0 Å compared to the corresponding experimental structures. In contrast, in the absence of the lipid, the antagonists display more diverse docking poses, resulting in a broader range of RMSD and ΔG_binding_ values (Fig. 5a, c). Among these, flipped poses are even more favored for ACT-709478 and TTA-A2 in the absence of the lipid (Fig. 5a). Our computational analyses support the role of the lipid in stabilizing the accommodation of the antagonists in the cavity, yet the physiological relevance and the identity and specificity of the endogenous lipids that may facilitate drug binding awaits further characterizations.

### Structural basis for ligand selectivity on T-type Ca_v_ channels

**Fig. 6:**
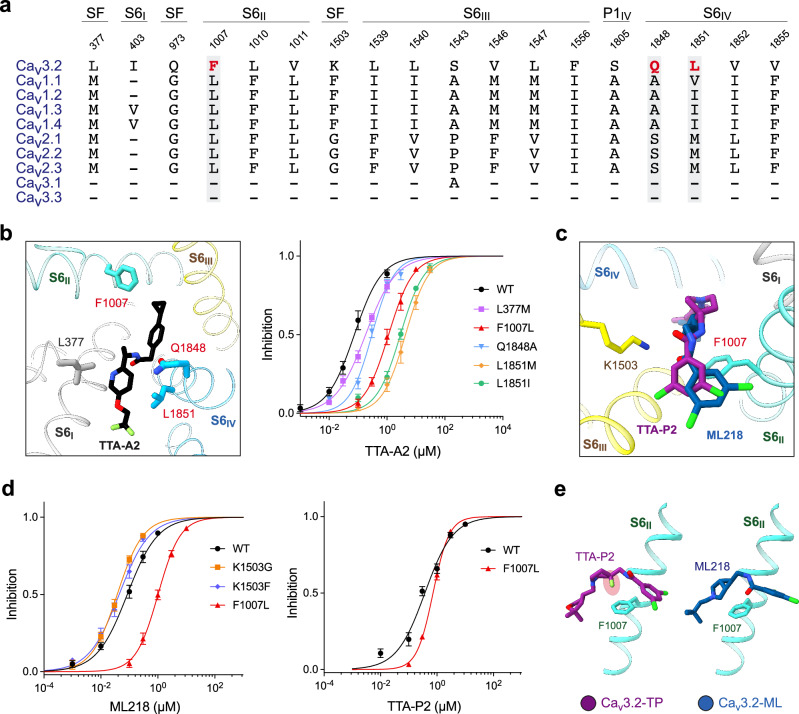
Structural basis for antagonist selectivity on T-type Ca_v_ channels. **a** Structure-guided sequence analysis to identify the determinants for antagonist selectivity. Sequence comparison of human Ca_v_ channels for the antagonist-binding residues is shown. The dashes represent residues in other subtypes that are identical to the corresponding ones in Ca_v_3.2. **b** Functional validation of residues critical to TTA-A2 selectivity. Several residues in Ca_v_3.2 were mutated to corresponding ones in the HVA channels. The responses of these mutants to TTA-A2 were examined through whole-cell patch-clamp recordings. **c** A magnified view of the coordination of TTA-P2 and ML218 in the superimposed structures of Ca_v_3.2-TP and Ca_v_3.2-ML. **d** F1007L confers Ca_v_3.2 resistance to ML218, but not TTA-P2. Experimental details are provided in Materials and Methods and Supplementary information, Figs. S9–S11, and Tables S3 and S4. **e** Potential molecular basis for the distinct responses to TTA-P2 and ML218 by Ca_v_3.2-F1007L. The additional fluorine atom in the piperidine ring of TTA-P2 may interfere with the essential π-H interaction between Phe1007 and the piperidine ring, potentially resulting in lower potency on Ca_v_3.2, which is largely unaffected by the Leu substitution.

The four structures presented here, along with our previously reported Ca_v_3.1-Z944 complex structure,^44^ offer important insights into T type-specific inhibition by these selective antagonists. Sequence alignment reveals that several residues involved in ligand binding, including Leu377, Gln973, Phe1007, Leu1010, Val1011, Lys1503, Leu1539, Leu1540, Ser1543, Val1546, Leu1547, Phe1556, Ser1805, Gln1848, Leu1851, Val1852, and Val1855, most of which are positioned on the P-loops and the S6 tetrahelical bundle, vary from those in the HVA Ca_v_ channels (Fig. 6a).

To identify the residues that underlie the subtype-specific sensitivity to these inhibitors, we started with in silico molecular docking and binding free energy calculation for Ca_v_3.2 mutants each with a single locus substituted with the corresponding residue from the HVA channels (Supplementary information, Fig. S9a). The computational analysis suggests that many mutations would have distinct impact on different inhibitors. For example, L377M, Q1848A/S, and L1851V/I/M resulted in decreased binding energy for ACT-709478 and TTA-A2, but not TTA-P2 or ML218. On the other hand, F1007L reduces the affinity to TTA-A2 and ML218, but has little effect on ACT-709478 and TTA-P2 (Supplementary information, Fig. S9).

Based on these clues, we generated a number of corresponding Ca_v_3.2 mutants and characterized their responses to the drugs using whole-cell patch-clamp recordings in HEK293T cells. Several mutations indeed attenuate the potency of the drugs, as exemplified by L377M, F1007L, Q1848A, L1851M and L1851I for TTA-A2, and F1007L for ML218. Consistent with the computational results, F1007L shows little effect on the potency of TTA-P2, which shares a similar binding pose to ML218 (Fig. 6b–d; Supplementary information, Figs. S9–S11 and Tables S3, S4).

The difference in F1007L’s responses to ML218 and TTA-P2 may be attributed to the additional fluorine atom in the piperidine ring of TTA-P2, which interferes with the π-H interaction between Phe1007 and the piperidine ring, leading to an ~30° deviation of the binding poses for the 3,5-dichlorobenzamide heads. Therefore, the potency of TTA-P2 on Ca_v_3.2, regardless of the F1007L mutation, is lower than that of ML218 (Fig. 6c–e; Supplementary information, Fig. S9, Table S4). As there is no fluorine atom in the piperidine group of Z944, an analog of ML218 and TTA-P2, we also performed similar analysis on the complex structure of Z944-bound Ca_v_3.1. Its overall binding pose in Ca_v_3.1 is closer to that of ML218 in Ca_v_3.2 than TTA-P2. Replacing the allelic Phe956 with Leu in Ca_v_3.1 significantly reduces the potency of Z944 on Ca_v_3.1 in a similar manner to that of ML218 on Ca_v_3.2,^44^ thereby confirming the distinct sensitivity of Phe1007 in Ca_v_3.2, or Phe956 in Ca_v_3.1, in ligand recognition (Supplementary information, Figs. S9–S11 and Table S4).

It is noted that K1503F or K1503G, which only slightly affects the binding energy, does not show a significant impact on the potency of ML218 on Ca_v_3.2 (Fig. 6d). A re-examination of the Z944 response by the corresponding Ca_v_3.1 mutants, K1462F or K1462G, reveals some technical issues in the previous electrophysiological characterizations, which are summarized in the legend of Supplementary information, Table S4. Using a corrected protocol, we show that K1462F or K1462G does not reduce Ca_v_3.1’s sensitivity to Z944 either (Supplementary information, Table S4).^44^

Taken together, our structural, computational, and functional analyses reveal the complexity underlying the determinants for the subtype specificity by selective inhibitors, and suggest that targeting these critical residues could be a viable strategy for designing selective T-type Ca_v_ channel blockers.

## Discussion

Given its pivotal role in epilepsy and pain, Ca_v_3.2 has emerged as a promising target for the development of anti-epileptics and analgesics.^39,55^ Dozens of pathological mutations, including those associated with autism, amyotrophic lateral sclerosis, hyperaldosteronism familial 4, and epilepsy, are directly linked to Ca_v_3.2 (Supplementary information, Table S5).^38,56–60^ The high-resolution structures of Ca_v_3.2 enable precise mapping of nineteen mutations located in the resolved regions. These mutations exhibit widespread distribution across various VSDs, ECLs, and the pore (Supplementary information, Fig. S12), establishing a structural foundation for understanding disease mechanisms.

**Fig. 7:**
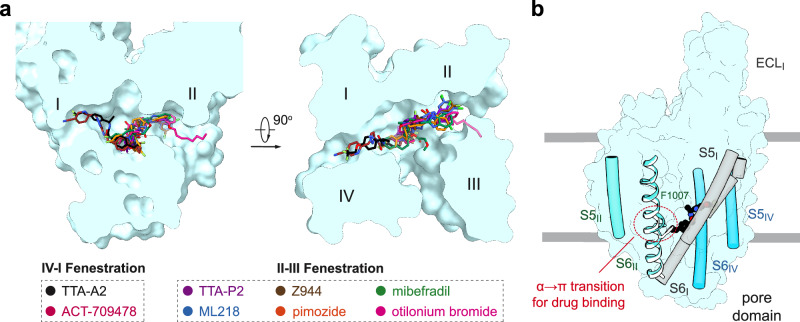
Structural mapping of drug-binding sites within the central cavity of T-type Ca_v_ channels. **a** Small-molecule T-type Ca_v_ blockers generally exhibit two binding poses within the cavity. Shown here are two perpendicular views of the superimposed PD of T-type Ca_v_ channels with inhibitors bound to the central cavity. TTA-A2 and ACT-709478 interact with the IV-I fenestration, while all others are associated with the II-III fenestration. **b** A common α-to-π transition of the S6_II_ segment when accommodating different antagonists. Among the various resolved structures of T-type Ca_v_ channels in complex with antagonists, ACT-709478 is the only one that binds to the α form.

The structures reported here and previously^44,47^ together reveal a common paradigm for T-type channel-selective pore blockers (Fig. 7a). These elongated molecules directly obstruct ion flow by occupying site C; meanwhile, the other end targets either site F2 (TTA-P2, ML218, Z944, pimozide, mibefradil, and otilonium bromide) or site F4 (ACT-709478 and TTA-A2) to stabilize the inactivated state. It is noted that the DHP compounds selectively target the III-IV fenestration (site F3) of Ca_v_1 channels, which are also known as the DHP receptors,^21^ while sites F2, F4, and C appear to be the promising regions in the T-type channels for developing selective blockers. Further chemical modifications of critical residues hold the promise to alter drug sensitivity and selectivity, providing opportunities for precise modulation of Ca_v_ subtypes.

Except Ca_v_3.2-ACT, which remains unchanged from the apo channel, a conserved α-to-π transition in the middle of the S6_II_ segment of all T-type channels is found in the presence of all other antagonists (Fig. 7b). Such secondary structural transition is also frequently observed in Na_v_ channels bound with pore blockers or even gating modifier toxins which bind to VSDs.^61^ The π-form S6 is usually associated with sealed fenestration(s), shrunk cavity volume, and contracted intracellular gate, physical features that all antagonize channel conductance. This observation suggests that the π-form structures might represent potentially more favored templates for Ca_v_- or Na_v_-targeting drug design.

Last but not least, small molecule interactions within the scaffold of the spacious cavity have been manifested by the drug-drug interaction between the antiviral sofosbuvir/MNI-1 and the anti-arhythmic amiodarone in the PD of Ca_v_1 channels.^15,62^ Endogenous lipids also represent an important class of small molecules. It is therefore not surprising that one or even more phospholipid molecules might interfere with antagonist binding by modifying the contour and chemical composition of the binding pocket. The role of lipids and other endogenous molecules, such as hormones and metabolites, in drug binding should also be investigated when targeting voltage-gated ion channels for drug discovery.

## Materials and methods

### Transient expression of human Ca_v_3.2 in HEK293F cells

The codon-optimized cDNA of human *CACNA1H* for Ca_v_3.2 isoform 1 (2353 residues, Uniprot Q95180-1) was synthesized by BGI Geneland Scientific Co., Ltd. To generate Ca_v_3.2EM, residues 493-772 were deleted using a standard two-step PCR method. Both the full-length Ca_v_3.2 and the truncated Ca_v_3.2EM were subcloned into the pCAG vector with Twin-Strep-tag and Flag-tag in tandem at the amino terminus. All the plasmids for transient expression were confirmed through DNA sequencing. HEK293F suspension cells (Thermo Fisher Scientific, R79007) were cultured in FreeStyle 293 medium (Thermo Fisher Scientific) at 37 °C, supplied with 5% CO_2_ under 60% humidity. Transfection of the cells with plasmids was carried out when the cell density reached 1.5–2.0 × 10^6^ cells/mL. For each 1-L cell culture, a mixture of 1.5 mg expression plasmids for Ca_v_3.2 and 3 mg 40-kDa linear polyethylenimines (Polysciences) in 50 mL fresh medium was incubated for 15–30 min, and then added to the cell culture to achieve transient expression of the human Ca_v_3.2 complex.

### Preparation of Ca_v_3.2 alone and drug-bound complexes

For one batch of protein purification, 15 L of transfected HEK293F cells were harvested ~48 h after transfection by centrifugation at 3600 × *g* for 10 min and resuspended in the lysis buffer containing 25 mM HEPES (pH 7.4), 150 mM NaCl, 2 mM CaCl_2_ and protease inhibitor cocktail (Selleckchem). After sonication on ice, the suspension was supplemented with n-dodecyl-β-D-maltopyranoside (Anatrace) to a final concentration of 1% (w/v), and cholesteryl hemisuccinate Tris salt (Anatrace) to 0.1% (w/v). After incubation at 4 °C for 2 h, the mixture was centrifuged at 16,000 × *g* for 45 min, and the supernatant was applied to anti-Flag M2 affinity gel (Sigma) for affinity purification. The resin was rinsed five times with the wash buffer (buffer W) that contains 25 mM HEPES (pH 7.4), 150 mM NaCl, 2 mM CaCl_2_, 0.06% GDN, and protease inhibitor cocktail. The target proteins were eluted with buffer W supplemented with 0.2 mg/mL Flag peptide (synthesized by GenScript). The eluent was concentrated using a 100-kDa cut-off Amicon filter unit (Millipore) and further purified through size-exclusion chromatography (Superose 6 10/300 GL, GE Healthcare) that was pre-equilibrated in the buffer containing 25 mM HEPES (pH 7.4), 150 mM NaCl, 2 mM CaCl_2_ and 0.02% GDN. Among the tested constructs of Ca_v_3.2, Ca_v_3.2EM afforded decent protein yield that was suitable for cryo-EM analysis. The peak fractions were pooled and concentrated to a final concentration of ~12 mg/mL.

To prepare drug-bound complexes, different drug (ACT-709478, TTA-A2, TTA-P2, or ML218) was added to concentrated Ca_v_3.2 proteins at the final concentration of 1 mM, and the mixtures were incubated at 4 °C for 45 min before preparation of cryo-EM grids.

### Cryo-EM sample preparation and data acquisition

The QuantiFoil grids (R1.2/1.3 300 mesh, Quantifoil) or UltrAuFoil grids (R1.2/1.3 300 mesh, Quantifoil) underwent glow-discharge using easiGlow (PELCO) at 15 mA for 15 s at 0.37 mBar. Prior to sample application, the Vitrobot Mark IV chamber was pre-cooled to 10 °C with 100% humidity. Subsequently, 3 μL of concentrated Ca_v_3.2-Apo/ACT/TA/TP/ML sample was applied to the freshly treated grid surface, followed by blotting with filter paper for 4 s. The grid was then swiftly plunged into liquid ethane and stored in liquid nitrogen.

For automated data collection, the grids were loaded onto a 300 kV Titan Krios G4 (Thermo Fisher) equipped with a Falcon IV detector (Thermo Fisher). Micrographs were captured using EPU (Thermo Fisher) in Falcon IV EC mode at a nominal magnification of 75,000 ×, resulting in a calibrated pixel size of 1.036 Å. Each movie stack in EER format was exposed for 6 s, accumulating a total electron dose of ~40 e^−^/Å2. Subsequently, the movie stacks underwent alignment, summation, and dose-weighting using cryoSPARC live.^63^

### Cryo-EM data processing

A total of 2,954/4,999/5,775/4,614/5,241 cryo-EM micrographs were collected for Ca_v_3.2-Apo/ACT/TA/TP/ML, respectively. During cryoSPARC live preprocessing, patched CTF estimation was implemented. For the Ca_v_3.2Apo dataset, 504 particles from 25 micrographs were manually picked to generate 2D templates through 2D classification. Subsequently, 1,812,275 particles were picked using the selected 2D templates through the template picker, and 2D classification was performed with bin-4 particles. 2D Class averages with different views were chosen as new references for the template picker. Two batches of picked particles underwent independent 2D classifications, and the well-classified particles were selected and merged. The Ca_v_3.1 structure (EMD-0791) was low-pass filtered and, together with a decoy junk density, used for heterogeneous refinement with bin-4 particles. The best class was selected and re-extracted to bin-2 for multiple rounds of continuous heterogeneous refinement. Subsequently, 565,821 particles were re-extracted into bin1 and subjected to another three rounds of heterogeneous refinement, resulting in a 3.4 Å NU-refinement result. This map was applied as a new heterogeneous refinement reference, considering higher frequency information. Through additional rounds of heterogeneous refinement and a final clean-up round of 2D classification, 103,429 particles were selected, leading to a reconstruction at 3.0 Å. A similar processing workflow was applied to other ligand-bound Ca_v_3.2 datasets, utilizing the apo-structure as the initial model.

### Model building and refinement

The Ca_v_3.2Apo initial model was automatically generated with ModelAngelo^64^ and subsequently subjected to manual examination and adjustments in COOT.^65^ Refinement against the corresponding map was then executed using the Real-space Refinement option in PHENIX.^66^ Additional structure optimization was conducted with ISOLDE,^67^ followed by a conclusive round of Real-space Refinement in PHENIX. The apo structure served as the new initial model for ligand-bound structures, employing the same model-building process. Detailed validation results for the model refinement are presented in Supplementary information, Table S2.

### Whole-cell electrophysiology

HEK293T cells were cultured in Dulbecco’s Modified Eagle Medium (BI) supplemented with 4.5 mg/mL glucose and 10% (v/v) fetal bovine serum (BI). For patch-clamp recordings, the cells were plated onto glass coverslips and transiently co-transfected with the Ca_v_3.2 variant plasmids and eGFP in the presence of lipofectamine 2000 (Invitrogen). Cells with green fluorescence were selected for patch-clamp recording 18–36 h after transfection. Experiments were performed at room temperature. No authentication was performed for the commercially available cell line. Mycoplasma contamination was not tested.

Whole-cell Ca_v_3.2 Ca^2+^ currents were recorded in HEK293T cells using an EPC10-USB amplifier with Patchmaster software v2*90.2 (HEKA Elektronik), filtered at 3 kHz (low-pass Bessel filter) and sampled at 50 kHz. The borosilicate pipettes (Sutter Instrument) used in all experiments had a resistance of 2–4 MΩ and series resistance was compensated by 70%–80%. The electrodes were filled with the internal solution composed of 125 mM CsCl, 10 mM EGTA, 2 mM CaCl_2_, 1 mM MgCl_2_, 5 mM Na_2_ATP, 10 mM HEPES, pH adjusted to 7.4 with CsOH. The bath solutions contained 5 mM CaCl_2_, 166 mM TEA-Cl, 10 mM HEPES, pH adjusted to 7.4 with TEA-OH. For the long-term drug delivery tests, the internal and external solutions were altered. The borosilicate pipettes were filled with a solution consisting of 130 mM Cs-methanesulfonate, 10 mM TEA-Cl, 10 mM EGTA, 5 mM MgCl_2_, 5 mM Na-ATP, 10 mM HEPES (pH 7.4 with CsOH) with a resistance of 2–4 MΩ, whereas the bath solutions contained 110 mM CsCl, 40 mM TEA-Cl, 5 mM BaCl_2_, 1 mM MgCl_2_, 10 mM _D_-glucose, and 10 mM HEPES (pH 7.4 with TEA-OH). Mutants with small currents were recorded with 10 mM BaCl_2_ in the external solution instead of 5 mM. Data were analyzed using Fitmaster 2.90.5 (HEKA Elektronik), Origin (OriginLab), and GraphPad Prism (GraphPad Software).

The voltage dependence of ion current (I-V) was analyzed using a protocol consisting of steps from a holding potential of –100 mV for 200 ms to voltages ranging from –90 mV to +50 mV for 150 ms in 5 mV increments. The linear component of leaky currents and capacitive transients were subtracted using the P/4 procedure. For activation property analysis, the equation G = I/(V – V_r_) was utilized, where V_r_, the reversal potential, represents the voltage at which the current is zero. For the activation curves, conductance (G) was normalized and plotted against the voltage from –90 mV to 0 mV or 20 mV. For voltage-dependent steady-state inactivation, cells were clamped at a holding potential of –100 mV and applied to step pre-pulses from –110 mV to –10 mV for 1000 ms with an increment of 5 mV. Subsequently, the Ca^2+^ currents were recorded at the test pulse of –30 mV for 100 ms. The peak currents under the test pulses were normalized and plotted against the prepulse voltage. Activation and inactivation curves were fitted with a Boltzmann function to determine V_1/2_ and slope values. The time course of inactivation data from the peak current at –30 mV was fitted with a single exponential equation: y = A_1_ exp(−x/τ_inac_) + y_0_, where A_1_ was the relative fraction of current inactivation, τ_inac_ was the time constant, x was the time, and y_0_ was the amplitude of the steady-state component.

To investigate the inhibition of Ca_v_3.2 variants by different drugs, cells were held at –100 mV and stepped to –30 mV for 150 ms. Drugs were dissolved in dimethyl sulfoxide (final concentration < 0.1%, Sigma) to make a stock solution. Solutions with the indicated concentrations were freshly prepared and perfused to the recording cell for up to several minutes to achieve the maximal blockade using a multichannel perfusion system (VM8, ALA). Prior to drug perfusion, cells were recorded for 5 min to establish a stable peak current. The concentration-response curve was fitted using the equation Y = Bottom + (Top – Bottom)/(1 + 10^(logIC50 – X) × Hill Slope^), where IC_50_ represented the concentration of the drugs that blocked 50% of the current and X denoted the log of drug concentration, and Hill Slope indicated the slope factor.

Data are presented as mean ± standard error of the mean (SEM), with *n* representing the number of experimental cells from which recordings were obtained. Statistical significance was assessed using an unpaired *t*-test with Welch’s correction, one-way ANOVA analysis, and extra sum-of-squares *F* test.

### Molecular docking and binding free energy calculation

The molecular docking analysis employed the Schrödinger Suite 2018-1 (Schrödinger, Inc.). Initial 3D configurations of small molecules were generated and optimized using the LigPrep program^68^ with the OPLS3 force field.^69^ The protein structure was prepared using default settings within Protein Preparation Wizard, utilizing the coordinates from the Ca_v_3.2–drug complexes. Subsequently, molecular docking simulations were executed with the extra-precision docking method (Glide XP) in the Glide program. The top-scored binding pose aligns well with the binding pose resolved in the cryo-EM structure and was selected for further analysis. Subtype-specific residues within the drug-binding site were mutated based on sequence alignment to those in other subtypes. The Prime-molecular mechanics/generalized Born surface area (MM/GBSA) method was employed to calculate the relative binding free energy changes (ΔΔG) for each mutant compared to the WT, while keeping the ligand and other residues fixed.

### Supplementary information

Supplementary information, Figure S1

Supplementary information, Figure S2

Supplementary information, Figure S3

Supplementary information, Figure S4

Supplementary information, Figure S5

Supplementary information, Figure S6

Supplementary information, Figure S7

Supplementary information, Figure S8

Supplementary information, Figure S9

Supplementary information, Figure S10

Supplementary information, Figure S11

Supplementary information, Figure S12

Supplementary information, Table S1

Supplementary information, Table S2

Supplementary information, Table S3

Supplementary information, Table S4

Supplementary information, Table S5

## Data Availability

The data that support this study are available from the corresponding authors upon reasonable request. The cryo-EM maps have been deposited in the Electron Microscopy Data Bank (EMDB) under accession codes EMD-43991 (Ca_v_3.2Apo), EMD-43995 (Ca_v_3.2-ACT), EMD-43992 (Ca_v_3.2-TA), EMD-43993 (Ca_v_3.2-TP), and EMD-43994 (Ca_v_3.2-ML). The coordinates have been deposited in the Protein Data Bank (PDB) under accession codes 9AYG (Ca_v_3.2Apo), 9AYL (Ca_v_3.2-ACT), 9AYH (Ca_v_3.2-TA), 9AYJ (Ca_v_3.2-TP), and 9AYK (Ca_v_3.2-ML).
