# Supplementary material for: Structural basis for human Cav3.2 inhibition by selective antagonists
Source: Cell Res. 2024 Apr 11;34(6):440–50. doi: 10.1038/s41422-024-00959-8 (PMC11143251; doi:10.1038/s41422-024-00959-8)
Supplement: Supplementary file 1 — Supplementary information, Figure S1 [file 41422_2024_959_MOESM1_ESM.pdf]

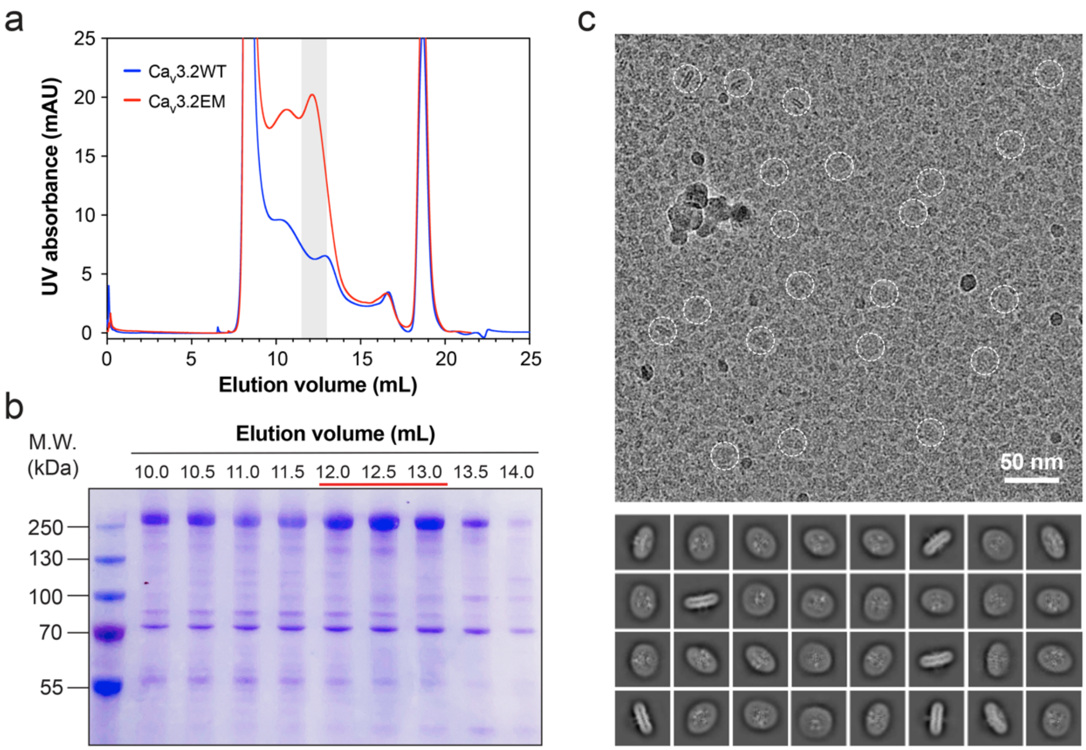

Supplementary information, Fig. S1. Purification and cryo-EM imaging of recombinant human Ca$_v$3.2. **a** Ca$_v$3.2EM exhibits better solution behavior than the wild-type (WT) protein. Shown here is a representative size-exclusion chromatography (SEC) purification for proteins obtained from 1 liter transfected HEK293F cells. Ca$_v$3.2EM: An engineered Ca$_v$3.2 construct with residues 493-772 deleted. **b** Purified Ca$_v$3.2EM for cryo-EM analysis. Shown here is a representative commassie blue-stained SDS-PAGE for last step purification through SEC. The peak fractions from large scale purification, highlighted with the red line and corresponding to the elution volumes shaded in the chromatogram above, were concentrated for cryo-EM sample preparation. **c** A representative cryo-EM micrograph (*up*) and 2D classifications (*down*) of Ca$_v$3.2EM. White circles indicate representative particles in distinct orientations.
