# Supplementary material for: Structural basis for human Cav3.2 inhibition by selective antagonists
Source: Cell Res. 2024 Apr 11;34(6):440–50. doi: 10.1038/s41422-024-00959-8 (PMC11143251; doi:10.1038/s41422-024-00959-8)
Supplement: Supplementary file 2 — Supplementary information, Figure S2 [file 41422_2024_959_MOESM2_ESM.pdf]

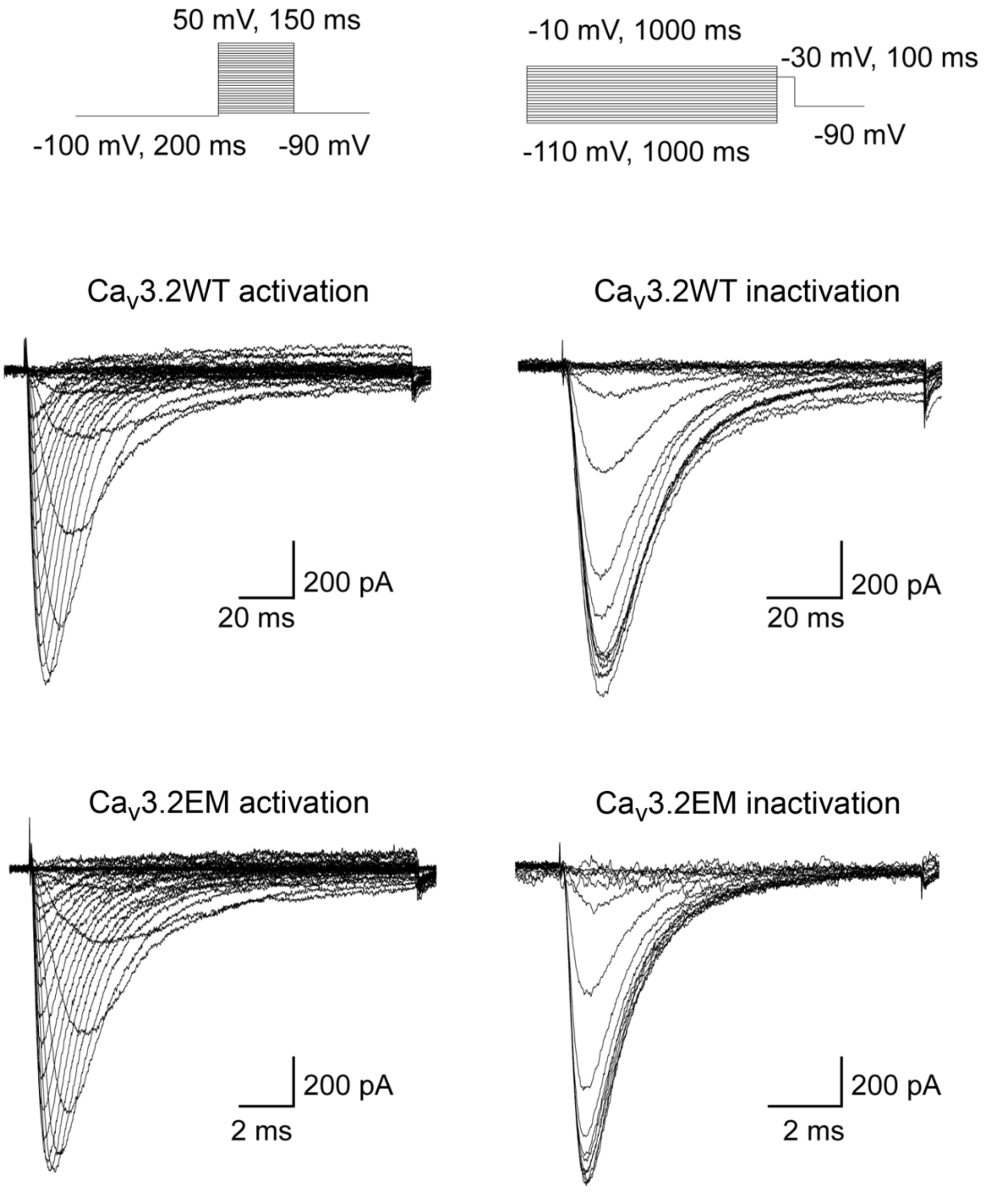

Supplementary information, Fig. S2. Electrophysiological recording of $Ca_v3.2$. Representative raw traces of the whole-cell patch clamp recordings for the activation and inactivation of full-length wild-type $Ca_v3.2$ ($Ca_v3.2$WT, *upper*) and truncated $Ca_v3.2$ construct that was used for cryo-EM analysis ($Ca_v3.2$EM, *lower*). Please refer to Methods and Supplementary information, Table S1 for experimental details.
