# Supplementary material for: Structural basis for human Cav3.2 inhibition by selective antagonists
Source: Cell Res. 2024 Apr 11;34(6):440–50. doi: 10.1038/s41422-024-00959-8 (PMC11143251; doi:10.1038/s41422-024-00959-8)
Supplement: Supplementary file 3 — Supplementary information, Figure S3 [file 41422_2024_959_MOESM3_ESM.pdf]

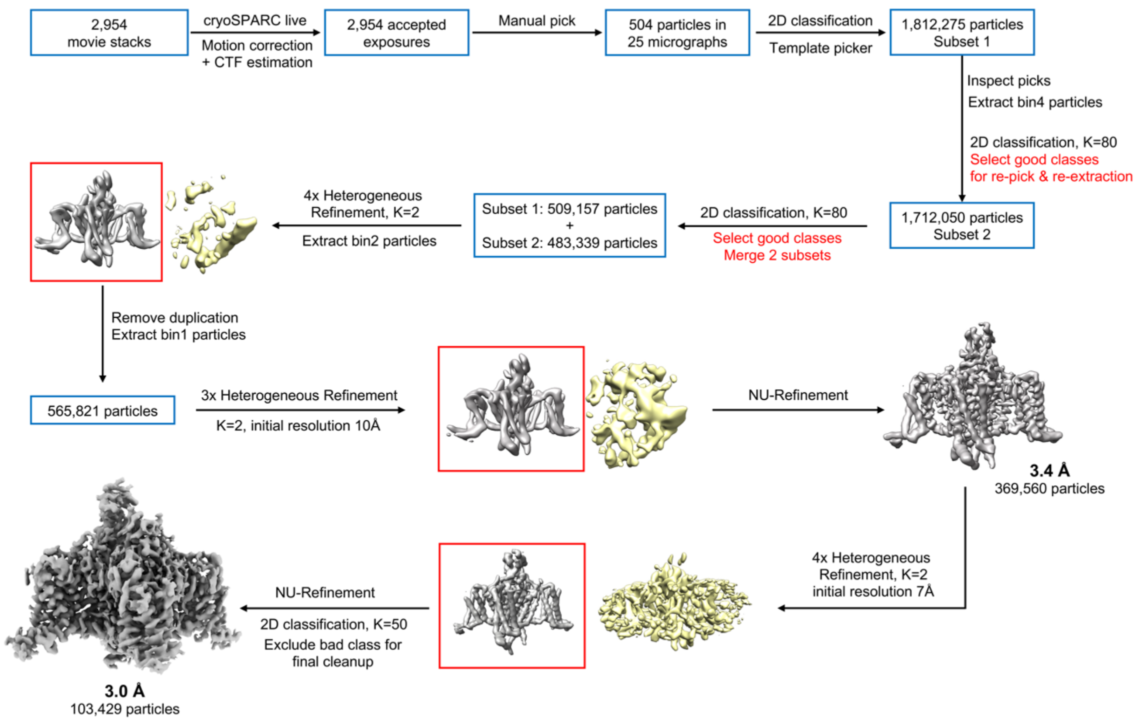

Supplementary information, Fig. S3. Flowchart for cryo-EM data processing.

Details can be found in Methods.
