# Supplementary material for: Structural basis for human Cav3.2 inhibition by selective antagonists
Source: Cell Res. 2024 Apr 11;34(6):440–50. doi: 10.1038/s41422-024-00959-8 (PMC11143251; doi:10.1038/s41422-024-00959-8)
Supplement: Supplementary file 4 — Supplementary information, Figure S4 [file 41422_2024_959_MOESM4_ESM.pdf]

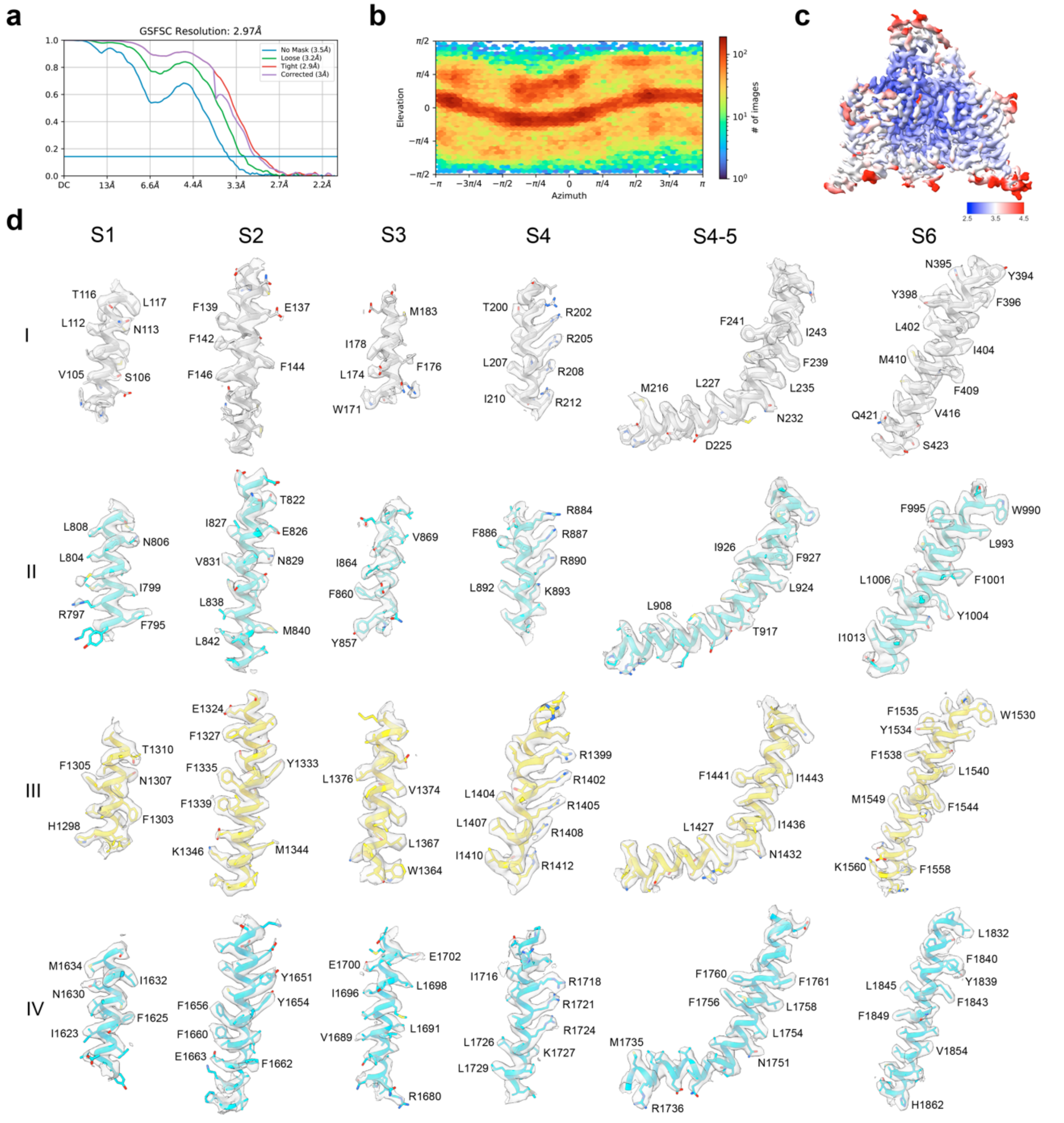

Supplementary information, Fig. S4. Cryo-EM analysis of Ca_v3.2Apo. **a** Gold standard fourier shell correlation (FSC) curves for the 3D reconstruction calculated in cryoSPARC[1]. **b** Angular distribution of particles for the final 3D reconstruction. **c** Local resolution distribution of the cryo-EM map for Ca_v3.2Apo. **d** Cryo-EM maps for the S1-S6 segments in each repeat. The densities, shown as semi-transparent gray surface, are contoured at 4-5 σ in ChimeraX[2].

References:

1 Punjani, A., Rubinstein, J. L., Fleet, D. J. & Brubaker, M. A. cryoSPARC: algorithms for rapid unsupervised cryo-EM structure determination. *Nat Methods* **14**, 290-296 (2017). https://doi.org/10.1038/nmeth.4169

2 Meng, E. C. *et al*. UCSF ChimeraX: Tools for structure building and analysis. *Protein Sci* **32**, e4792 (2023). https://doi.org/10.1002/pro.4792
