# Supplementary material for: Structural basis for human Cav3.2 inhibition by selective antagonists
Source: Cell Res. 2024 Apr 11;34(6):440–50. doi: 10.1038/s41422-024-00959-8 (PMC11143251; doi:10.1038/s41422-024-00959-8)
Supplement: Supplementary file 5 — Supplementary information, Figure S5 [file 41422_2024_959_MOESM5_ESM.pdf]

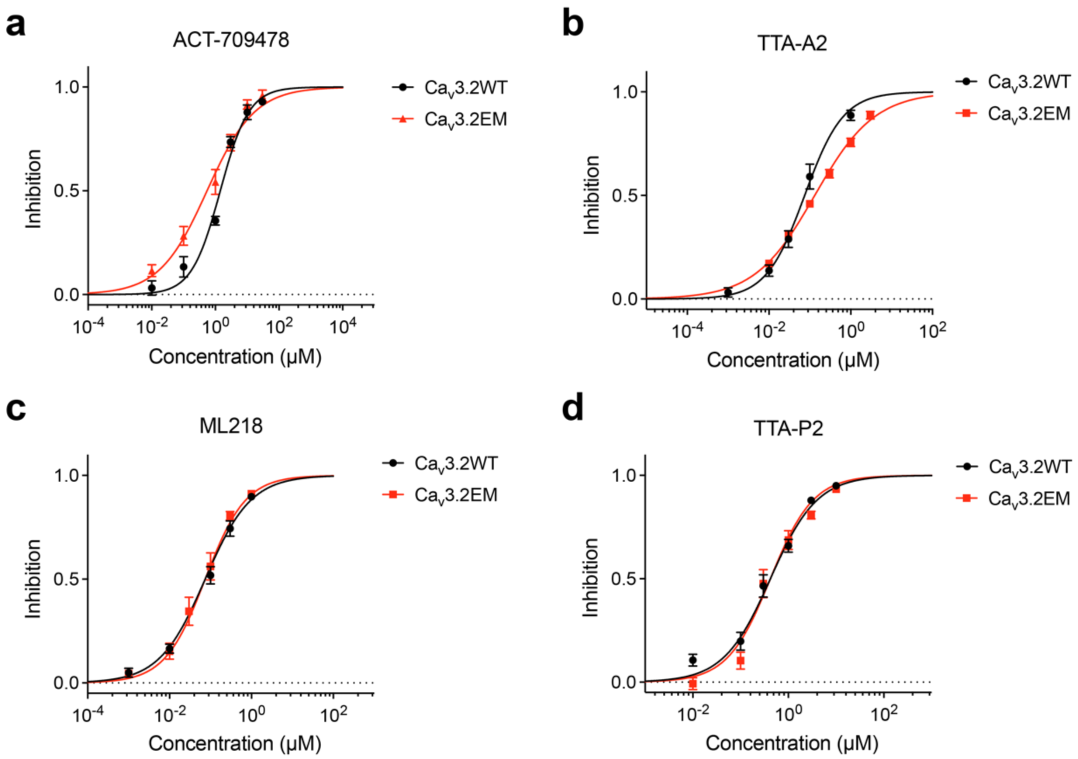

Supplementary information, Fig. S5. Dose-dependent inhibition of Ca$_v$3.2 by four T-type selective antagonists. Ca$_v$3.2EM and Ca$_v$3.2WT exhibit comparable responses to all tested antagonists, including ACT-709478 (**a**), TTA-A2 (**b**), ML218 (**c**), and TTA-P2 (**d**). Please refer to Supplementary information, Table S4 for details of the IC$_{50}$ measurements.
