# Supplementary material for: Structural basis for human Cav3.2 inhibition by selective antagonists
Source: Cell Res. 2024 Apr 11;34(6):440–50. doi: 10.1038/s41422-024-00959-8 (PMC11143251; doi:10.1038/s41422-024-00959-8)
Supplement: Supplementary file 6 — Supplementary information, Figure S6 [file 41422_2024_959_MOESM6_ESM.pdf]

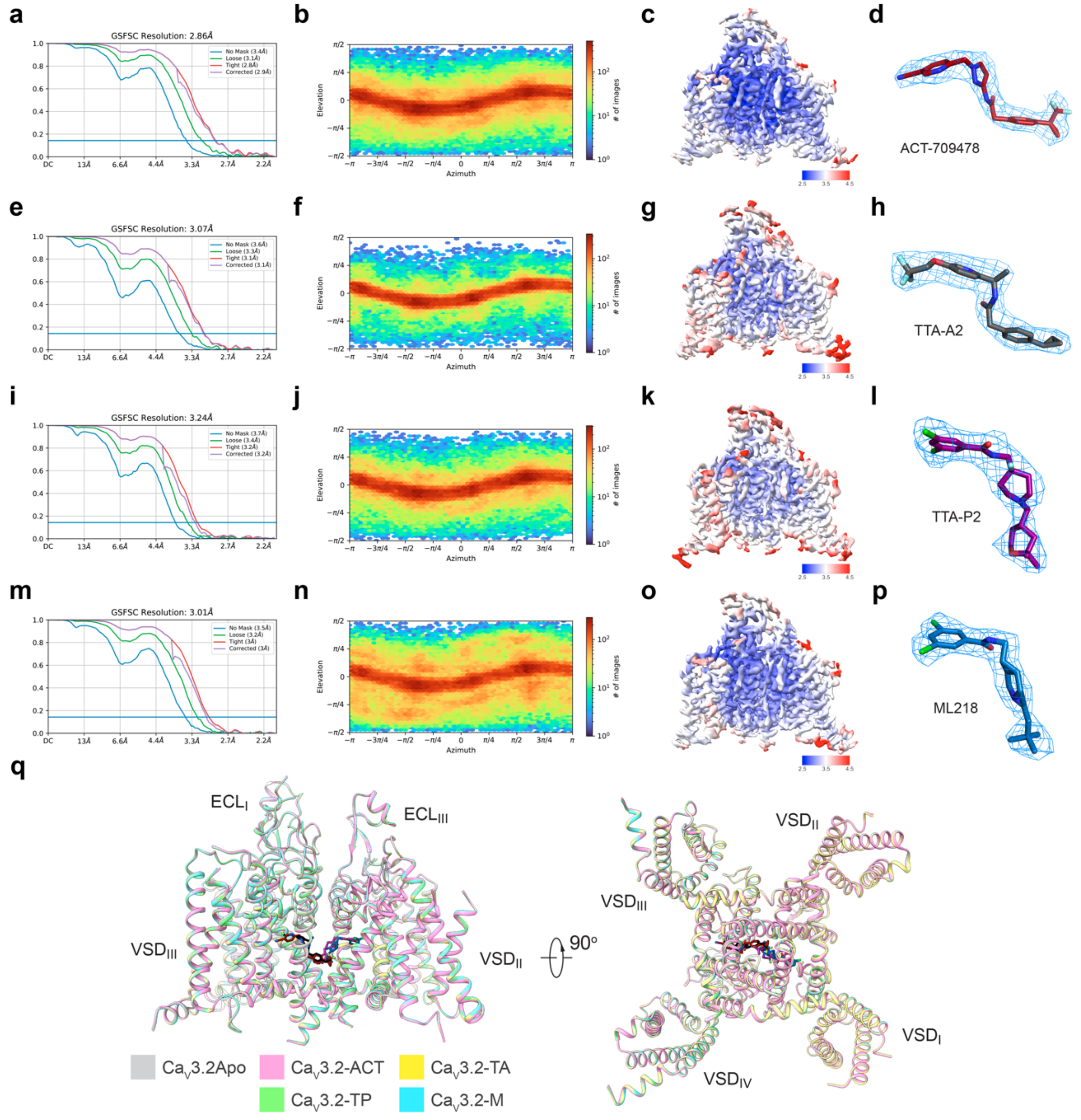

Supplementary information, Fig. S6. Cryo-EM analysis of $Ca_v3.2$-antagonist complexes. Gold standard fourier shell correlation (FSC) curves, angular distribution of particles, and local resolution distribution of the cryo-EM map are shown respectively in panels **a-c** for $Ca_v3.2$-ACT, **e-g** for $Ca_v3.2$-TA, **i-k** for $Ca_v3.2$-TP, and **m-o** for $Ca_v3.2$-ML. Cryo-EM densities for ACT-709489, TTA-A2, TTA-P2, and ML218, all contoured at 5 σ in Pymol[1], are respectively shown in panels **d**, **h**, **i**, and **p**. **q** $Ca_v3.2$Apo and antagonist-bound complexes all exhibit similar overall structures. Shown here are two perpendicular views of the superimposed structures.

References

1 The PyMOL Molecular Graphics System, Version 3.0 (Schrödinger, Inc.).
