# Supplementary material for: Structural basis for human Cav3.2 inhibition by selective antagonists
Source: Cell Res. 2024 Apr 11;34(6):440–50. doi: 10.1038/s41422-024-00959-8 (PMC11143251; doi:10.1038/s41422-024-00959-8)
Supplement: Supplementary file 7 — Supplementary information, Figure S7 [file 41422_2024_959_MOESM7_ESM.pdf]

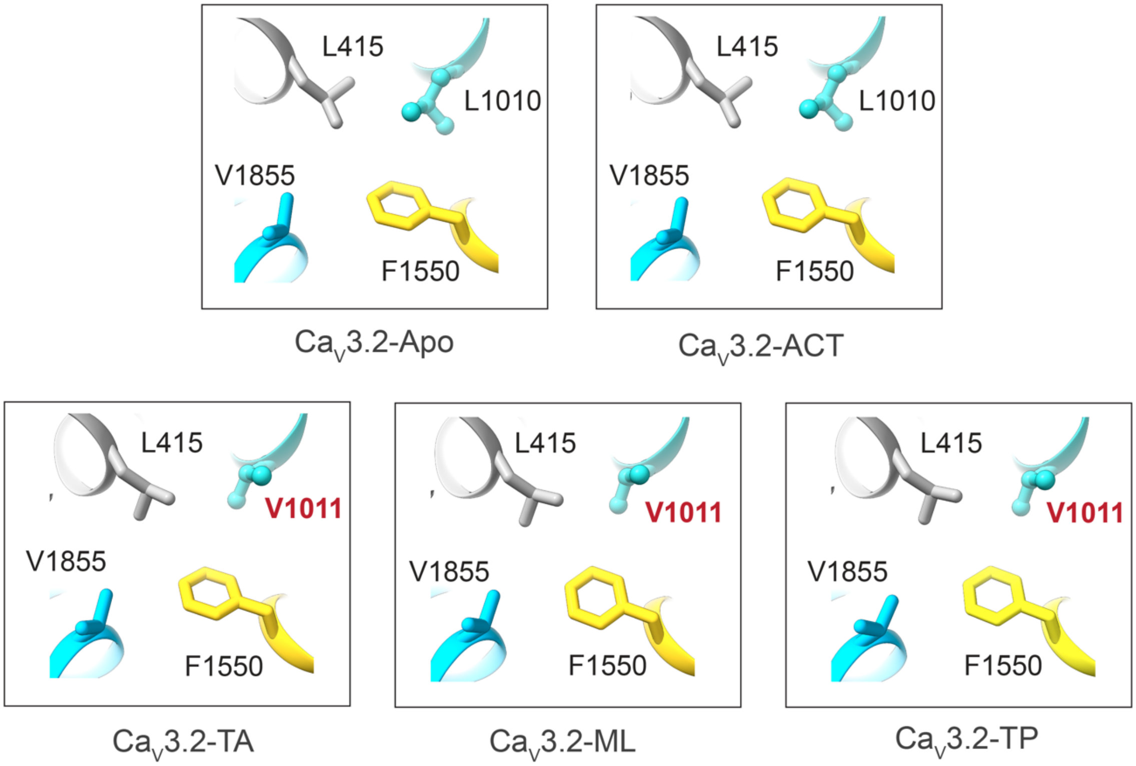


**Supplementary information, Fig. S7. Structural shifts of the gating residues accompanying the α→π transition upon antagonist binding.** Shown here are identical extracellular views of the gating residues in the indicated $Ca_v3.2$ structures. Except ACT-709478, binding of the other antagonists leads to a shift of one gating residue from Leu1010 to Val1011 accompanying the α→π transition of the $S6_{II}$ segment.
