# Supplementary material for: Structural basis for human Cav3.2 inhibition by selective antagonists
Source: Cell Res. 2024 Apr 11;34(6):440–50. doi: 10.1038/s41422-024-00959-8 (PMC11143251; doi:10.1038/s41422-024-00959-8)
Supplement: Supplementary file 8 — Supplementary information, Figure S8 [file 41422_2024_959_MOESM8_ESM.pdf]

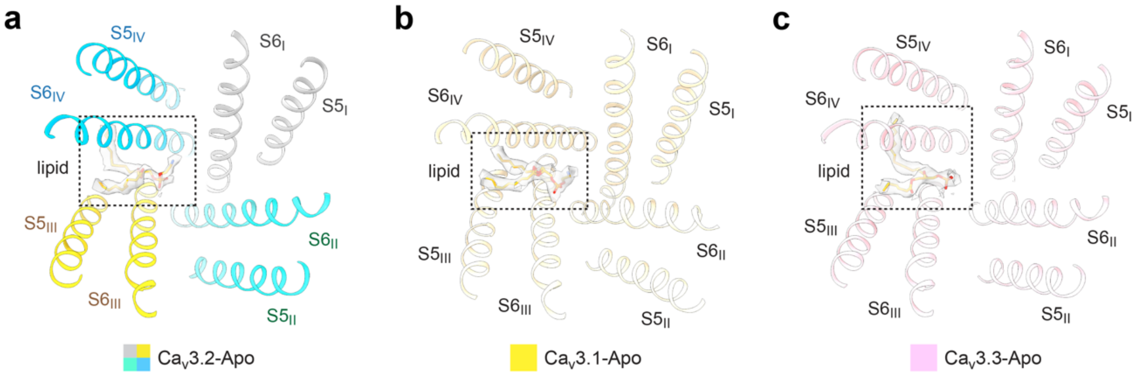

Supplementary information, Fig. S8. An endogenous lipid consistently binds to the III-IV fenestrations of all T-type calcium channels (TTCCs). Shown here are the same extracellular views of the pore domain from each of the TTCC subtypes, adopted from the apo structures of human $Ca_v3.2$, $Ca_v3.1$ (PDB: 6KZO, EMDB: EMD-0791), and $Ca_v3.3$ (PDB: 7WLI, EMDB: EMD-32584). Cryo-EM densities for the endogenous lipid in each structure are depicted as semi-transparent gray surface and contoured at 4.5-5 σ in ChimeraX[1].

References:

1 Meng, E. C. *et al*. UCSF ChimeraX: Tools for structure building and analysis. *Protein Sci* **32**, e4792 (2023). https://doi.org/10.1002/pro.4792
