# Supplementary material for: Structural basis for human Cav3.2 inhibition by selective antagonists
Source: Cell Res. 2024 Apr 11;34(6):440–50. doi: 10.1038/s41422-024-00959-8 (PMC11143251; doi:10.1038/s41422-024-00959-8)
Supplement: Supplementary file 9 — Supplementary information, Figure S9 [file 41422_2024_959_MOESM9_ESM.pdf]

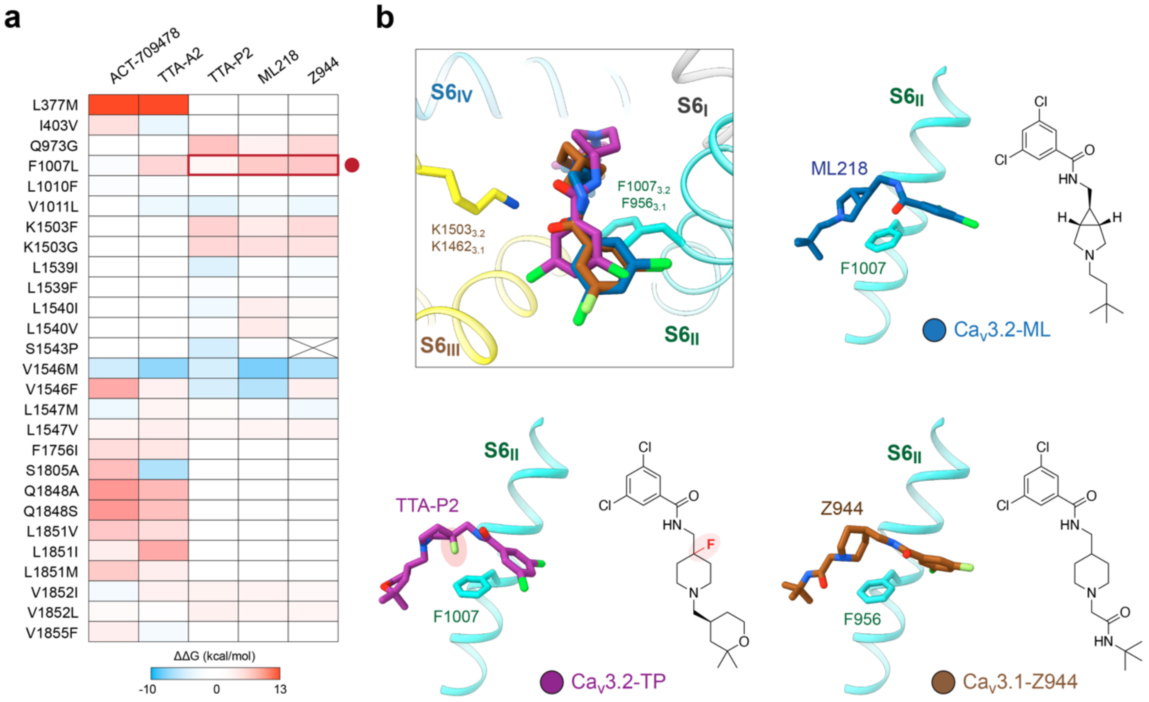

Supplementary information, Fig. S9. Molecular basis for the subtype-specific responses to the antagonists. **a** Computational evaluation of the residue contribution to antagonist binding. Residues in $Ca_v3.2$ channels are substituted with corresponding residues in the high-voltage-activated (HVA) channels, and the subsequent change in binding free energy (ΔG) is calculated using the Prime-MM/GBSA method. The corresponding mutations for Z944 binding in $Ca_v3.1$ are evaluated accordingly. A positive ΔΔG value (red) indicates that such mutation leads to less favored interaction with the indicated antagonist. **b** The binding pose of Z944 in $Ca_v3.1$ is more similar to that of ML218 in $Ca_v3.2$ than TTA-P2. Structures of $Ca_v3.2$-TP, $Ca_v3.2$-ML, and $Ca_v3.1$-Z944 (PDB: 6KZP) are superimposed relative to their pore domain. All three antagonists are presented as sticks. The channel structure is shown for $Ca_v3.2$-TP only for visual clarity. The mutation F1007L, which confers resistance to TTA-A2 (Fig. 6b) and ML218 (Fig. 6d), has little effect on $Ca_v3.2$'s sensitivity to TTA-P2 (Fig. 6e). The distinct antagonist responses to F1007L in $Ca_v3.2$ (equivalent of F956L in $Ca_v3.1$) could be attributed to the additional fluorine in the piperidine ring of TTA-P2, which may potentially interfere with the essential π-H interaction with Phe1007.
