# Supplementary material for: Structural basis for human Cav3.2 inhibition by selective antagonists
Source: Cell Res. 2024 Apr 11;34(6):440–50. doi: 10.1038/s41422-024-00959-8 (PMC11143251; doi:10.1038/s41422-024-00959-8)
Supplement: Supplementary file 10 — Supplementary information, Figure S10 [file 41422_2024_959_MOESM10_ESM.pdf]

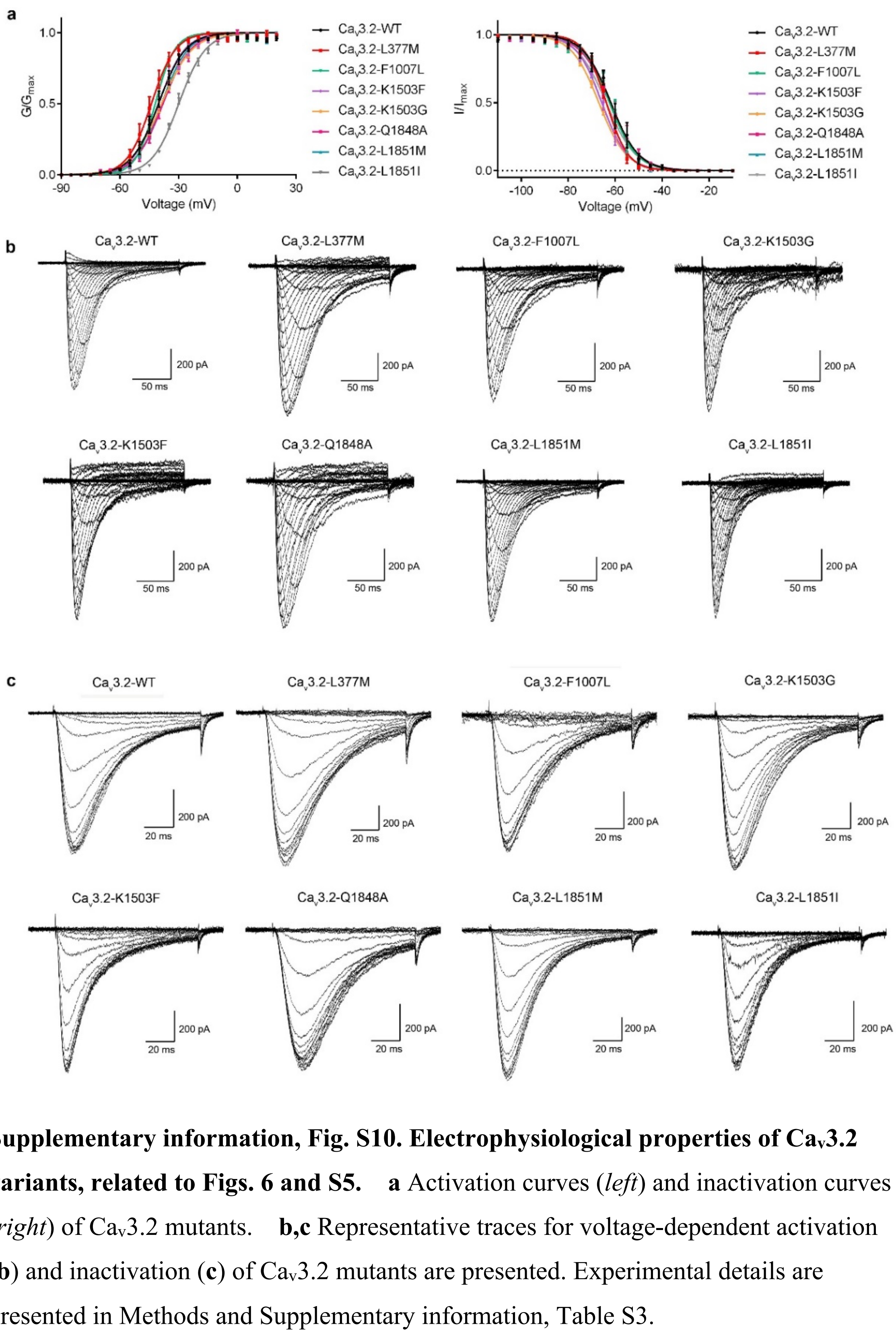

Supplementary information, Fig. S10. Electrophysiological properties of Ca$_v$3.2 variants, related to Figs. 6 and S5. **a** Activation curves (*left*) and inactivation curves (*right*) of Ca$_v$3.2 mutants. **b,c** Representative traces for voltage-dependent activation (**b**) and inactivation (**c**) of Ca$_v$3.2 mutants are presented. Experimental details are presented in Methods and Supplementary information, Table S3.
