# Supplementary material for: Structural basis for human Cav3.2 inhibition by selective antagonists
Source: Cell Res. 2024 Apr 11;34(6):440–50. doi: 10.1038/s41422-024-00959-8 (PMC11143251; doi:10.1038/s41422-024-00959-8)
Supplement: Supplementary file 11 — Supplementary information, Figure S11 [file 41422_2024_959_MOESM11_ESM.pdf]

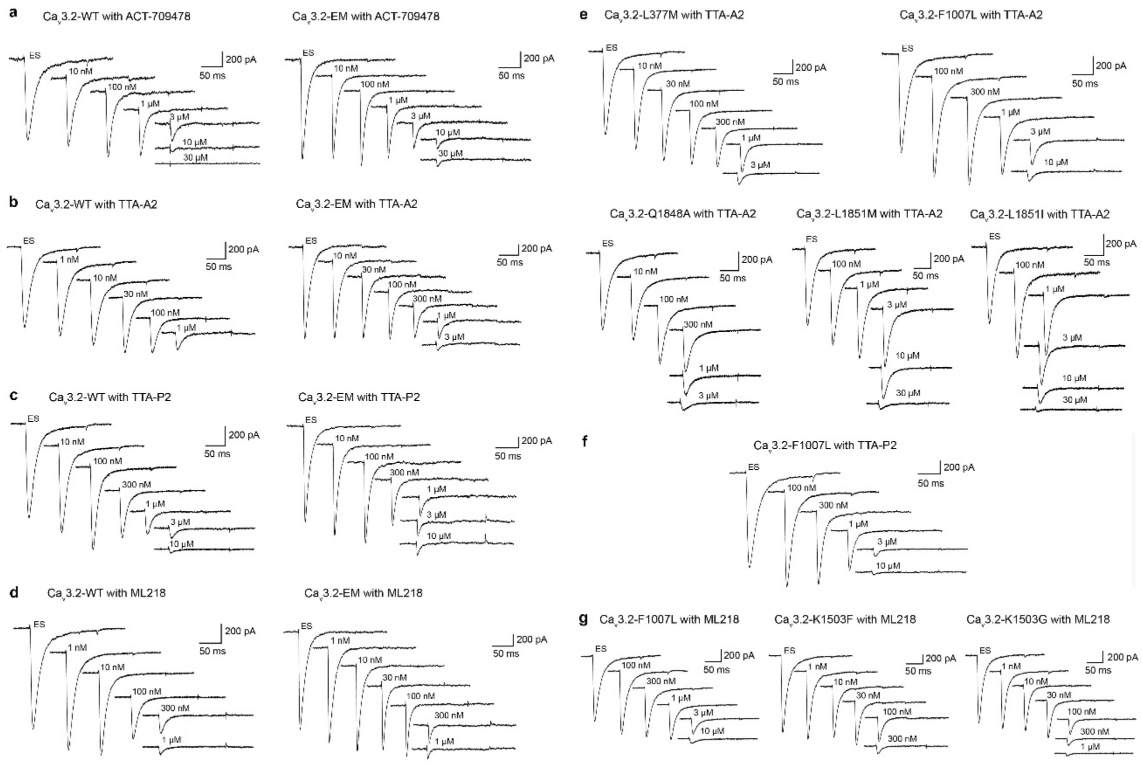

Supplementary information, Fig. S11. Block of Ca$_V$3.2 variants by indicated drugs, related to Figs. 6, S5, and S10. **a-d** Representative traces for blocking Ca$_V$3.2WT and Ca$_V$3.2EM with ACT-709478 (**a**), TTA-A2 (**b**), TTA-P2 (**c**), and ML218 (**d**) at indicated concentrations. **e-g** Representative traces for blocking Ca$_V$3.2-mutants with TTA-A2 (**e**), TTA-P2 (**f**), and ML218 (**g**) at indicated concentrations. Experimental details are presented in Methods and Supplementary information, Table S4.
