# Supplementary material for: Structural basis for human Cav3.2 inhibition by selective antagonists
Source: Cell Res. 2024 Apr 11;34(6):440–50. doi: 10.1038/s41422-024-00959-8 (PMC11143251; doi:10.1038/s41422-024-00959-8)
Supplement: Supplementary file 12 — Supplementary information, Figure S12 [file 41422_2024_959_MOESM12_ESM.pdf]

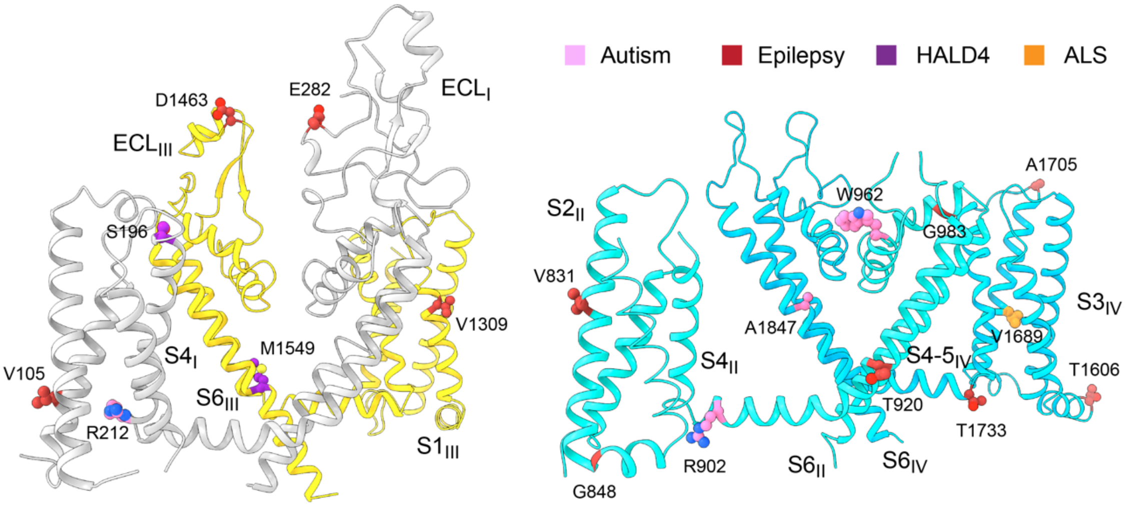

Supplementary information, Fig. S12. Structural mapping of disease-related mutations identified in Ca$_v$3.2. Please refer to Supplementary information, Table S5 for details. Two side views of the diagonal repeats are shown. HALD4: Hyperaldosteronism, familial, 4. ALS: Amyotrophic lateral sclerosis.
