# Supplementary material for: Structural basis for human Cav3.2 inhibition by selective antagonists
Source: Cell Res. 2024 Apr 11;34(6):440–50. doi: 10.1038/s41422-024-00959-8 (PMC11143251; doi:10.1038/s41422-024-00959-8)
Supplement: Supplementary file 13 — Supplementary information, Table S1 [file 41422_2024_959_MOESM13_ESM.pdf]

Supplementary information, Table S1. Activation and steady-state inactivation parameters of $Ca_v3.2$WT and $Ca_v3.2$EM in HEK293T cells, related to Fig. 1a.

	Parameters	$Ca_v3.2$WT	$Ca_v3.2$EM
**Activation**	$V_{1/2}$ (mV)	-42.96 ± 0.34	-50.50 ± 0.71****
	P	/	< 0.0001
	slope	5.88 ± 0.30	6.55 ± 0.62
	P	/	0.2810
	n	12	6
**Inactivation**	$V_{1/2}$ (mV)	-59.84 ± 0.31	-66.62 ± 0.48****
	P	/	< 0.0001
	slope	-5.47 ± 0.27	-6.17 ± 0.42
	P	/	0.1951
	τ_{inac} (ms)	20.71 ± 0.86	19.65 ± 1.71
	P	/	0.5518
	n	13	9

**** P < 0.0001 versus WT. Each data point represents mean ± s.e.m (standard deviation of mean) and *n* is the number of experimental cells from which recordings were obtained. The extra sum-of-squares F test was used to compare the $V_{1/2}$ of activation and inactivation fits. τ_{inac} values of $Ca_v3.2$WT and $Ca_v3.2$EM inactivation were compared by using an unpaired t-test with Welch's correction.
