# Supplementary material for: Structural basis for human Cav3.2 inhibition by selective antagonists
Source: Cell Res. 2024 Apr 11;34(6):440–50. doi: 10.1038/s41422-024-00959-8 (PMC11143251; doi:10.1038/s41422-024-00959-8)
Supplement: Supplementary file 14 — Supplementary information, Table S2 [file 41422_2024_959_MOESM14_ESM.pdf]

Supplementary information, Table S2. Statistics for data collection and structural refinement.

	$Ca_v3.2$Apo	$Ca_v3.2$-TA	$Ca_v3.2$-TP	$Ca_v3.2$-ML	$Ca_v3.2$-ACT
**Data collection and processing**					
Magnification	75,000	75,000	75,000	75,000	75,000
Voltage (kV)	300	300	300	300	300
Electron dose (e-/Å^2)	50	50	50	50	50
Defocus range (μm)	-1.5~-1.0	-1.5~-1.0	-1.5~-1.0	-1.5~-1.0	-1.5~-1.0
Pixel size (Å)	1.036	1.036	1.036	1.036	1.036
Symmetry	C1	C1	C1	C1	C1
Initial particle images	1,712,050	3,241,117	3,734,266	4,315,540	4,194,215
Final particle images	103,429	89,595	113,396	126,670	188,950
Map resolution (Å)	3.0/3.1	3.1/3.2	3.2/3.3	3.0/3.1	2.8/3.0
FSC threshold (half-map/model-map)	0.143/0.5	0.143/0.5	0.143/0.5	0.143/0.5	0.143/0.5
**Refinement**					
Initial model used	-	apo	apo	apo	apo
Map sharpening *B* factor (Å^2)	-77.8	-79.0	-100.0	-93.4	-97.1
Model composition					
Non-hydrogen atoms	8822	8854	8855	8851	8853
Protein residues	1055	1056	1056	1056	1065
Ligands	12	13	13	13	13
B factors (Å^2)					
Protein	62.17	72.23	75.37	66.45	82.78
Ligand	23.61	69.69	68.04	61.41	70.64
R.m.s deviations					
Bond lengths (Å)	0.004	0.002	0.002	0.002	0.003
Bond angles (°)	0.601	0.465	0.496	0.481	0.519
Validation					
MolProbity score	1.00	1.20	1.23	1.09	1.07
Clashscore	2.23	4.11	4.56	3.00	2.84
Poor rotamers (%)	0.32	0	0.21	0.75	0.96
Ramachandran plot					
Favored (%)	98.07	98.27	98.36	98.46	98.75
Allowed (%)	1.93	1.73	1.64	1.54	1.25
Disallowed (%)	0.00	0.00	0.00	0.00	0.00
