# Supplementary material for: Structural basis for human Cav3.2 inhibition by selective antagonists
Source: Cell Res. 2024 Apr 11;34(6):440–50. doi: 10.1038/s41422-024-00959-8 (PMC11143251; doi:10.1038/s41422-024-00959-8)
Supplement: Supplementary file 15 — Supplementary information, Table S3 [file 41422_2024_959_MOESM15_ESM.pdf]

Supplementary information, Table S3. Activation and steady-state inactivation parameters of Ca$_v$3.2 variants in HEK293T cells, related to Supplementary information, Fig. S10.

	Parameters	Ca$_v$3.2-WT	L377M	F1007L	K1503F	K1503G	Q1848A	L1851M	L1851I
**Activation**	$V_{1/2}$ (mV)	-40.32 ± 0.36	-43.98 ± 0.43****	-42.0 ± 0.22****	-40.29 ± 0.42	-38.76 ± 0.28**	-38.05 ± 0.38****	-38.70 ± 0.28**	-29.96 ± 0.21****
	P	/	< 0.0001	< 0.0001	0.9647	0.0031	< 0.0001	0.0010	< 0.0001
	slope	6.907 ± 0.32	6.17 ± 0.38	4.98 ± 0.19****	6.67 ± 0.37	7.83 ± 0.25*	7.10 ± 0.33	6.72 ± 0.25	6.82 ± 0.19****
	P	/	0.1451	< 0.0001	0.6341	0.0442	0.6919	0.6701	0.8568
	n	11	7	12	8	7	8	8	6
**Inactivation**	$V_{1/2}$ (mV)	-61.86 ± 0.48	-63.78 ± 0.26***	-62.45 ± 0.41	-65.69 ± 0.30****	-66.97 ± 0.16****	-62.41 ± 0.50	-62.27 ± 0.45	-63.87 ± 0.38**
	P	/	0.0005	0.3663	< 0.0001	< 0.0001	0.4248	0.5425	0.0018
	slope	-5.90 ± 0.40	-4.70 ± 0.23*	-5.79 ± 0.38	-5.45 ± 0.27	-5.86 ± 0.14	-6.23 ± 0.46	-5.81 ± 0.42	-6.34 ± 0.33
	P	/	0.0134	0.8470	0.3758	0.9245	0.6148	0.8784	0.4411
	τ_{inac} (ms)	26.82 ± 2.31	29.91 ± 3.76	33.75 ± 2.55	20.31 ± 1.98	39.09 ± 3.87	35.75 ± 5.00	40.00 ± 7.42	24.63 ± 1.38
	P	/	0.9910	0.4961	0.6872	0.0970	0.3537	0.0864	0.9994
	n	7	6	10	6	6	6	5	5

* P < 0.05 versus WT, ** P < 0.01 versus WT, *** P < 0.001 versus WT, **** P < 0.0001 versus WT. Each data point represents mean ± s.e.m (standard deviation of mean) and n is the number of experimental cells from which recordings were obtained. The extra sum-of-squares F test was used to compare the $V_{1/2}$ of activation and inactivation fits. τ_{inac} values of Ca$_v$3.2-WT and Ca$_v$3.2-mutations inactivation were compared by using one-way ANOVA analysis.
