# Supplementary material for: Structural basis for human Cav3.2 inhibition by selective antagonists
Source: Cell Res. 2024 Apr 11;34(6):440–50. doi: 10.1038/s41422-024-00959-8 (PMC11143251; doi:10.1038/s41422-024-00959-8)
Supplement: Supplementary file 16 — Supplementary information, Table S4 [file 41422_2024_959_MOESM16_ESM.pdf]

Supplementary information, Table S4. Characterizations of $Ca_v3.2$ residues that confer subtype-specific sensitivity to TTA-A2.

		Ca_v3.2WT	Ca_v3.2EM	L377M	F1007L	Q1848A	L1851M	L1851I
IC_{50} (μM)		0.0746 ± 0.0089	0.1313 ± 0.0082**	0.1823 ± 0.0156****	1.135 ± 0.1041****	0.3026 ± 0.0255****	4.347 ± 0.4378****	2.912 ± 0.2260****
P		/	0.0015	< 0.0001	< 0.0001	< 0.0001	< 0.0001	< 0.0001
Slope		0.9205 ± 0.1229	0.5902 ± 0.0236**	0.8298 ± 0.0633	1.028 ± 0.0979	1.133 ± 0.1044	1.081 ± 0.1174	1.050 ± 0.0857
P		/	0.0011	0.5145	0.5158	0.2382	0.3763	0.4554
n	1 nM	3	/	/	/	/	/	/
	10 nM	5	6	6	6	9	/	/
	30 nM	11	5	6	5	/	/	/
	100 nM	7	5	6	6	10	5	5
	300 nM	/	5	5	6	6	/	/
	1 μM	6	6	5	5	8	6	5
	3 μM	/	6	/	/	8	5	5
	10 μM	/	/	/	/	/	6	5
	30 μM	/	/	/	/	/	5	5

Phe1007, but not Lys1503, contributes to $Ca_v3.2$'s subtype-specific sensitivity to ML218.

		$Ca_v3.2$WT	$Ca_v3.2$EM	K1503G	K1503F	F1007L
IC_{50} (μM)		0.0846 ± 0.0090	0.0678 ± 0.0080	0.0374 ± 0.0027****	0.0438 ± 0.0043****	1.016 ± 0.0644****
P		/	0.2129	< 0.0001	< 0.0001	< 0.0001
Slope		0.8093 ± 0.0734	0.8730 ± 0.1029	0.9564 ± 0.0661	0.8132 ± 0.0745	1.113 ± 0.0748**
P		/	0.6202	0.1630	0.9708	0.0073
n	1 nM	5	2	5	5	/
	10 nM	6	5	7	6	/
	30 nM	/	6	5	6	/
	100 nM	9	7	7	5	7
	300 nM	6	6	5	5	7
	1 μM	7	5	/	/	7
	3 μM	/	/	/	/	6
	10 μM	/	/	/	/	7

Phe1007 has little impact on the Ca_v3-specific sensitivity to TTA-P2.

TTA-P2		Ca_v3.2WT	Ca_v3.2EM	F1007L
IC_{50} (μM)		0.3709 ± 0.0333	0.4392 ± 0.0499	0.7226 ± 0.0317****
P		/	0.2867	< 0.0001
Slope		0.8659 ± 0.0732	0.9689 ± 0.1243	1.513± 0.0902****
P		/	0.4572	< 0.0001
	10 nM	5	5	/
	100 nM	9	6	5
	300 nM	8	8	5
	1 μM	8	9	6
	3 μM	8	5	5
	10 μM	5	5	5

ACT-709478		Ca_v3.2WT	Ca_v3.2EM
IC_{50} (μM)		1.427 ± 0.1479	0.5476± 0.0883****
P		/	< 0.0001
Slope		0.9659± 0.1075	0.5790 ± 0.0622**
P		/	0.0033
	10 nM	8	9
	100 nM	7	7
	1 μM	7	8
	3 μM	5	7
	10 μM	5	4
	30 μM	5	2

** P < 0.01 versus WT, **** P < 0.0001 versus WT. Each data point represents mean ± s.e.m (standard deviation of mean), and *n* is the number of experimental cells from which recordings were obtained. The extra sum-of-squares F test was used to compare the IC_{50} and slope factor of concentration-response curves.

Lys1503 in human Ca_v3.2 corresponds to Lys1462 in human Ca_v3.1. We previously reported that substitution of Ca_v3.1-Lys1462 (utilizing the Ca_v3.1-Δ8b construct) with Phe or Gly reduced the sensitivity to Z944 inhibition[1]. By repeating the experiments in both Yan lab and Lory lab, we found that the protocol used by the co-authors in the previous electrophysiological characterizations had the following technical issues. 1) Inappropriate recording protocols during the perfusion test. Several minutes after the whole-cell recording was established, the cells were kept recording at 1 Hz. Z944 was perfused continuously from low to high concentrations; each concentration was perfused for ~ 30 s in most cases, and only the first 20 pulses were analyzed. The duration of the perfusion might be insufficient for the action of Z944. In addition, the patch-clamp status,

such as the seal resistance and series resistance, could not be monitored or adjusted during continuous recording. 2) Large leak currents. The seal resistance of nearly half of cells for $Ca_v3.1$-Δ8b and K1462F perfusion test was not large enough, a sign of large leak currents that might lead to inaccurate peak currents. 3) The series resistance was not compensated well, especially for $Ca_v3.1$-Δ8b and K1462F, which usually generated large currents (>2 nA in half of cells). The series resistance after compensation was larger than 10 MΩ in all cells for $Ca_v3.1$-Δ8b and half of the cells for K1462F. 4) With the above issues, the SEM should be large if it were not due to the data processing protocol, wherein the pulses from all cells under the same concentration were pooled as a group to fit a single exponential function and calculate the average inhibition rate. In fact, recordings from each cell should be analyzed individually. Being aware of these problems, we have redesigned recording and analysis protocols. As the V_{max} of $Ca_v3.1$ was around -35 mV, we adjusted the test pulse from -20 mV to -30 mV for $Ca_v3.1$-Δ8b-K1462G/K1462F or -40 mV for $Ca_v3.1$-Δ8b-F956L. The series resistance was ~5-10 MΩ and compensated with ~80-95%. Only cells with large seal resistance were used. We perfused the recorded cells with increasing Z944 concentrations for several minutes until achieving maximal block, then acquired three continuous recordings for average to calculate the inhibition percentage. Recordings from each cell were analyzed individually. With the new protocol, mutations of K1462 did not significantly alter the IC_{50} of Z944. Our further investigation revealed that Phe956, which corresponds to Phe1007 in $Ca_v3.2$, is the key residue conferring T-type specificity for Z944.

References:

1 Zhao, Y. *et al*. Cryo-EM structures of apo and antagonist-bound human Ca(v)3.1. *Nature* **576**, 492-497 (2019). https://doi.org/10.1038/s41586-019-1801-3
