# Supplementary material for: Structural basis for human Cav3.2 inhibition by selective antagonists
Source: Cell Res. 2024 Apr 11;34(6):440–50. doi: 10.1038/s41422-024-00959-8 (PMC11143251; doi:10.1038/s41422-024-00959-8)
Supplement: Supplementary file 17 — Supplementary information, Table S5 [file 41422_2024_959_MOESM17_ESM.pdf]

**Supplementary information, Table S5. Disease-related mutations mapped to the $Ca_v$3.2 structure, related to Supplementary information, Fig. S12.**

| Mutations | Disease | Structure | Mutations | Disease | Structure |
|---|---|---|---|---|---|
| R212C | Autism[1] | $S4_{I}$ | G755D | Epilepsy | I-II linker |
| R902W | Autism[1] | $S4\text{-}5_{II}$ | G773D | Epilepsy | I-II linker |
| W962C | Autism[1] | $P1_{II}$ | G784S | Epilepsy | I-II linker |
| A1847V | Autism[1] | $S6_{IV}$ | V831M | Epilepsy | $S2_{II}$ |
| S196L | HALD4 | $S4_{I}$ | G848S | Epilepsy | $S2_{II}$ |
| M1549I | HALD4 | $S6_{III}$ | A876T | Epilepsy[2] | $S3\text{-}4_{II}$ |
| M1549V | HALD4 | $S6_{III}$ | T920M | Epilepsy[2] | $S5_{II}$ |
| P2083L | HALD4 | CTD | G983S | Epilepsy[2] | $P2_{II}$ |
| P1210L | ALS[3] | II-III linker | A1059S | Epilepsy[2] | II-III linker |
| V1689M | ALS[4] | $S3_{IV}$ | E1170K | Epilepsy[2] | II-III linker |
| A1705T | ALS[4] | $S3\text{-}4_{IV}$ | Q1264H | Epilepsy[2] | II-III linker |
| V105G | Epilepsy[2] | $S1_{I}$ | V1309I | Epilepsy[2] | $S1_{III}$ |
| F161L | Epilepsy | $S2\text{-}3_{I}$ | D1463N | Epilepsy | $ECL_{III}$ |
| Q163H | Epilepsy[2] | $S2\text{-}3_{I}$ | T1606M | Epilepsy[2] | $S0_{IV}$ |
| E282K | Epilepsy | $ECL_{I}$ | A1705T | Epilepsy[2] | $S3\text{-}4_{IV}$ |
| A332T | Epilepsy[2] | $ECL_{I}$ | T1733A | Epilepsy[2] | $S4\text{-}5_{IV}$ |
| C456S | Epilepsy | I-II linker | R1892H | Epilepsy[2] | CTD |
| A480T | Epilepsy[5] | I-II linker | A1966V | Epilepsy[2] | CTD |
| G499S | Epilepsy | I-II linker | R2005C | Epilepsy[2] | CTD |
| P618L | Epilepsy | I-II linker | A2140T | Epilepsy[2] | CTD |
| P648L | Epilepsy | I-II linker | A2170T | Epilepsy[2] | CTD |
| R744Q | Epilepsy | I-II linker | M2312V | Epilepsy[2] | CTD |
| A748V | Epilepsy | I-II linker | | | |

**HALD4**: Hyperaldosteronism, familial, 4; **ALS**: Amyotrophic lateral sclerosis.

Disease mutations in structurally unresolved regions are shaded light gray. Mutations are summarized from https://www.uniprot.org/uniprotkb/O95180/ and references[1-5].

**References:**

1 Splawski, I. *et al*. CACNA1H mutations in autism spectrum disorders. *J Biol Chem* **281**, 22085-22091 (2006). https://doi.org/10.1074/jbc.M603316200

2 Heron, S. E. *et al*. Extended spectrum of idiopathic generalized epilepsies associated with CACNA1H functional variants. *Ann Neurol* **62**, 560-568 (2007). https://doi.org/10.1002/ana.21169

3 Stringer, R. N. *et al*. A rare CACNA1H variant associated with amyotrophic lateral sclerosis causes complete loss of Ca(v)3.2 T-type channel activity. *Mol Brain* **13**, 33 (2020). https://doi.org/10.1186/s13041-020-00577-6

4 Rzhepetskyy, Y., Lazniewska, J., Blesneac, I., Pamphlett, R. & Weiss, N. CACNA1H missense mutations associated with amyotrophic lateral sclerosis alter Cav3.2 T-type calcium channel activity and reticular thalamic neuron firing. *Channels (Austin)* **10**, 466-477 (2016). https://doi.org/10.1080/19336950.2016.1204497

5 Heron, S. E. *et al*. Genetic variation of CACNA1H in idiopathic generalized epilepsy. *Ann Neurol* **55**, 595-596 (2004). https://doi.org/10.1002/ana.20028
